# A systematic review of emergency department based HIV testing and linkage to care initiatives in low resource settings

**DOI:** 10.1371/journal.pone.0187443

**Published:** 2017-11-02

**Authors:** Bhakti Hansoti, Gabor D. Kelen, Thomas C. Quinn, Madeleine M. Whalen, Taylor T DesRosiers, Steven J. Reynolds, Andrew Redd, Richard E. Rothman

**Affiliations:** 1 Department of Emergency Medicine, Johns Hopkins University School of Medicine, Baltimore, Maryland, United States of America; 2 Division of Intramural Research, NIAID/NIH, Baltimore, Maryland, United States of America; Thai Red Cross AIDS Research Centre, THAILAND

## Abstract

**Introduction:**

Only 45% of people currently living with HIV infection in sub-Saharan Africa are aware of their HIV status. Unmet testing needs may be addressed by utilizing the Emergency Department (ED) as an innovative testing venue in low and middle-income countries (LMICs). The purpose of this review is to examine the burden of HIV infection described in EDs in LMICs, with a focus on summarizing the implementation of various ED-based HIV testing strategies.

**Methodology and results:**

We performed a systematic review of Pubmed, Embase, Scopus, Web of Science and the Cochrane Library on June 12, 2016. A three-concept search was employed with emergency medicine (e.g., Emergency department, emergency medical services), HIV/AIDS (e.g., human immunodeficiency virus, acquired immunodeficiency syndrome), and LMIC terms (e.g., developing country, under developed countries, specific country names).

The search returned 2026 unique articles. Of these, thirteen met inclusion criteria and were included in the final review. There was a large variation in the reported prevalence of HIV infection in the ED population ranging from to 2.14% in India to 43.3% in Uganda. The proportion HIV positive patients with previously undiagnosed infection ranged from 90% to 65.22%.

**Conclusion:**

In the United States ED-based HIV testing strategies have been front and center at curbing the HIV epidemic. The limited number of ED-based studies we observed in this study may represent the paucity of HIV testing in this venue in LMICs. All of the studies in this review demonstrated a high prevalence of HIV infection in the ED and an extraordinarily high percentage of previously undiagnosed HIV infection. Although the numbers of published reports are few, these diverse studies imply that in HIV endemic low resource settings EDs carry a large burden of undiagnosed HIV infections and may offer a unique testing venue.

## Introduction

Despite significant strides in combating HIV worldwide, the AIDS pandemic continues. The UNAIDS Gap Report declares that it will be impossible to end the epidemic without bringing HIV treatment to all who need it [1]. Consequently, UNAIDS has adopted ambitious treatment targets called “90-90-90” that stipulate by 2010 90% of those infected with HIV will be aware of their diagnosis, of which 90% will be successful linked to acre (LTC) and of which 90% will achieve viral suppression [2].

Significant challenges lie ahead with regard to reaching these goals. Currently there are 22 million people worldwide who either do not have access to life-saving treatment due to coverage gaps within the health care system [2, 3]. Globally the HIV epidemic is concentrated, with 15 countries accounting for nearly 75% of all individuals living with HIV. Two thirds of these countries are located in sub-Saharan Africa (South Africa, Nigeria, Kenya, Mozambique, Uganda, Tanzania, Zimbabwe, Zambia, Malawi, Ethiopia); the remaining five countries include India, China, Russia, Brazil, and the United States [1]. Many of these countries (all except Russia and the USA) fall under the definition of low-income economies (either designated as low-income countries (LICs) (less than $1,045 USD GNI per capita) or lower-middle income countries (LMICs) ($1,045-$4,125 USD GNI per capita) as defined by the World Bank [4]. It is in these economies where healthcare resources are limited, that the number of people with unknown HIV infection, new HIV infections, and AIDS related deaths remain the highest [5].

Early detection of undiagnosed HIV infection with subsequent effective HIV treatment is widely recognized to extend life expectancy, improves life quality, and reduces HIV transmission, making it a cost-effective public health intervention [3]. Accordingly, the key to early treatment is early recognition with the first stage of the HIV care continuum (or “cascade”) beginning with diagnosis [6]. Unfortunately, only 45% of people currently living with HIV in sub-Saharan Africa are aware of their HIV status (about half of the UNAIDS goal) [5]. While promising results have emerged from innovative mobile and home testing initiatives to identify key/under-tested populations a wider sustained testing strategy is required in venues that provides care to key populations and are able to effectively integrate HIV screening into routine care [7].

The Emergency Department (ED) provides care large volumes of individuals who present for episodic care, many of whom use emergency care services as their sole source of care [8]. In the United States (US), HIV testing was expanded to non-traditional venues such as EDs and non-clinical settings. This highly successful strategy has made significant strides in curbing the HIV epidemic in the US [9]. The rise of integrated HIV testing into US emergency departments (which serve over 140 million patients/year) has been shown to parallel declines in rates undiagnosed HIV infection, and increases in rates of antiretroviral use [9, 10]. The ED is now recognized by the Centers for Disease Control to be central to the national HIV testing strategy, and the implementation of ED-based HIV screening programs is recommended by the US Preventive Services Task Force (USPSTF) when the local prevalence of HIV infection is >0.1% and has been adopted by the American College of Emergency Physicians [11–13]. As ED-based HIV testing has gathered momentum, numerous studies have also sought to understand the best approaches to implementing HIV testing in the ED for example opt-in versus opt-out approaches, it is unclear what strategies are feasible in low resource EDs [14] [15].

While there is, strong evidence supporting the role and impact of ED-based HIV testing in the US, there is a dearth of knowledge regarding what the scope of missed HIV infection may be in EDs across low resource settings. The purpose of this review is to quantify the burden of HIV infection described in EDs in low resource settings and examine the acceptance and feasibility of ED-based HIV testing strategies in LMICs.

## Materials and methods

**Fig 1:**
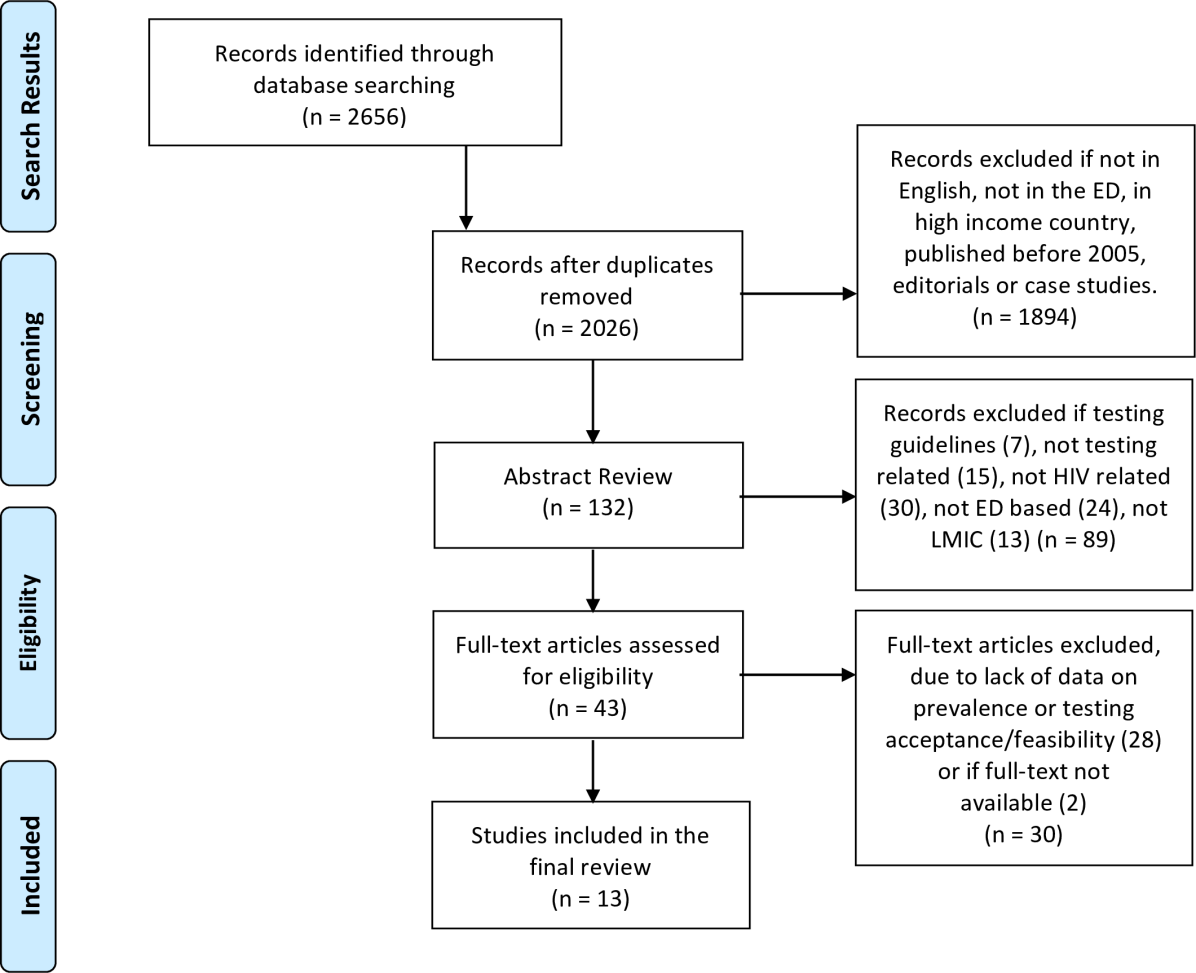
PRISMA flow diagram of articles included in the systematic review.

We performed an initial search of Pubmed, Embase, Scopus, Web of Science and the Cochrane Library on June 12^th^ 2016 with an updated search on January 1^st^ 2017. A three-concept search was employed with emergency medicine (e.g., Emergency department, emergency medical services), HIV/AIDS (e.g., human immunodeficiency virus, acquired immunodeficiency syndrome), and low and middle-income country terms (e.g., developing country, under developed countries, and specific country names employing the Cochrane Collaboration's low and middle-income filter) [16]. A full search strategy is presented in S1 Appendix. All applicable controlled vocabularies and keyword terms were searched. The concepts for the search strategy were developed in collaboration with a professional librarian (KL) and clinicians (BH, TD, SH). The search was run without any restrictions and two authors screened each result. Only articles published from Jan 1^st^ 2005 to Jan 1^st^ 2017 were included. Studies were included if they described a specific ED-based testing strategy, presented data on HIV prevalence in the ED, or presented data on the acceptability/feasibility of an ED-based testing program. Studies were excluded if they were conducted in high income settings, were purely descriptive, editorials, or case based studies (defined as an n less than 5. A final breakdown of our search strategy is presented in Fig 1. Authors further evaluated the studies for data on HIV prevalence (including undiagnosed), LMIC status, and ED-based testing strategies. Two independent authors reviewed each study with identified discrepancies resolved by a third senior author (BH).

## Results

The search returned 2026 unique articles. Of these, thirteen articles met inclusion criteria and were included in the final review. Three studies were in LICs [17–19], nine in LMICs [20–29]. Seven studies examined adult populations, one study examined both adult and pediatric populations, and two studies examined only the pediatric population. A summary of all the adult studies included in the review are provided in Table 1 and pediatric studies included in the review are provided in Table 2.

**Table 1:** Summary of all adult studies included in the systematic review and reported HIV prevalence, proportion of undiagnosed HIV infection and the testing strategy utilized.

Quantitative Studies including adult patients										
Country	Year	Journal	Author	Type of study	Tested (N)/total offered	Sampling Method	Point of Testing	Total HIV + (%)	Total new HIV diagnosis (%)	Type of HIV test
India	2010	Ind J of STDs	Devi [26]	Cross sectional	400	Unclear	Unclear	23 (5.75%)	15 (65.22%)	COMB AIDS-RS (HIV 1 and 2 Immunodot test kit) confirmed by Retroqic HIV and HIV Tridot for HIV I/II antibodies
India^++^	2010	Indian J Pathol Microbiol	Minz [25]	Cross sectional	607	Symptoms of HIV (Targeted)	Pediatric, Surgical, and Medical EDs	13 (2.14%)	Unknown	Rapid Kits (Signal HIV test, Comb AIDS test, Immunocomb II HIV test). Confirmed using micro-ELISA.
India	2014	Int J of STD and AIDS	Minz [24]	Retrospe-ctive review	654	Targeted	ED	30 (4.60%)	Unknown	4th generation micro-ELISA, confirmation w/ GENEDIA and TRIDOT
Uganda*	2006 & 2007	Afr Health Sci	Nakan- jako [17, 19]	Cross sectional	198/233	Every 6th Patient	Medical ED	86 (43.40%)	86 (77.00%)	3 sequential rapid tests; Determine, HIV ½ Stat-Pak, Unigold.
Kenya	2007	EMJ	Ranney [21]	Retrospe-ctive review	285/321	Sexually assaulted women	ED	7 (2.46%)	5 (71.40%)	not defined
India	2004	Indian J Med Microbiol.	Teja [22]	Retrospe-ctive review	1187	Known HIV positive	Inpatient	Unknown	90%	not defined
India	2008	Indian J Med Microbiol.	Teja [23]	Retrospe-ctive review	10752	Provider initiated (Targeted)	ED (medical patients only)	317 (2.90%)	211 (84.75%)	Rapid HIV for emergent surgery (HIV Tri dot) + ELISA for all Vironostika HIV ag/ab Combi, reactive specimens reevaluated by 2nd assay; discordants confirmed by 4th Gen VIDAS HIV Ultra Duo
Kenya	2007	AIDS Patient Care and STDs	Wax -man [20]	Retrospe-ctive review	1339/ 1371	Symptoms of HIV, Opt-out testing	Medical ED	312 (22.70%)	Unknown	Two rapid tests: Uni-Gold^TM^ Recombigen^(R)^ and Determine^(R)^ HIV-1/2

* 2 papers by Nakajanko et al. present data from the same dataset

** 419 patients (25.7% had no HIV result documented in the chart)

**Table 2:** Summary of all pediatric studies included in the systematic review and reported HIV prevalence, proportion of undiagnosed HIV infection and the testing strategy utilized.

Country	Year	Journal	Author	Type of study	Tested (N)/total offered	Sampling Method	Point of Testing	Total HIV + (%)	Total new HIV diagnosis (%)	Type of HIV test
Malawi*	2010	EMJ	Ahmad [18]	Cross sectional	576	Critically ill children only	ED	152 (26%)	Unknown	Bedside antibody test for HIV ½, confirmed by HIV RNA PCR.
India**	2010	Ind J of Path and Micro	Minz [25]	Cross sectional	239	Symptoms of HIV (Targeted)	ED	13 (2.14%)	Unknown	Rapid Kits (Signal HIV test, Comb AIDS test, Immunocomb. HIV test). Confirmed w/ micro-ELISA.
Tanz -ania	2016	BMJ Open	Sawe [29]	Retrospe-ctive review	1632/ 5540	Charts reviewed	ED and Inpatient	239 (14.5%)**	Unknown	not defined

* In this study children under 18 months excluded

** Same article presents data on both adults and children, thus has been included twice in the table above

### HIV prevalence

Nine studies quantified the burden of HIV infection in ED patients [17, 18, 20, 21, 23–26, 29]. The countries included in this review were: India [22–26], Kenya [20, 21, 28], Uganda [17, 19], Malawi [18], Tanzania [29] and Guyana [27]. There was a large variation in the reported prevalence of HIV infection in the ED population ranging from to 2.14% in India to 43.3% in Uganda [19, 25]. Only five studies were able to provide data on the burden of undiagnosed HIV infection [17, 21–23, 26]. The burden of previously undiagnosed infection reported ranged from 90% to 65.22% [23, 26].

### ED-based HIV testing strategies

The majority of studies (6) were conducted as retrospective chart reviews of all ED patients that presented for care during the study period, only four studies attempted to perform a cross-sectional prevalence study, i.e., they implemented a testing strategy over a defined period and then assessed testing acceptance and reactivity rates.[17–19, 25, 26] Two of the studies used fourth generation lab based testing and conducted on samples already collected from patients, both of these studies were also conducted in India [23, 25, 26]. The remaining studies that report testing strategies used rapid point of care tests with confirmatory ELISA or PCR [17–19, 28].

### Acceptance and feasibility of ED-based HIV testing

In four studies, it was possible to calculate the proportion of patients who accepted HIV testing when offered. In two studies the testing strategy was also defined, Waxman et al. offered opt-out testing and Nakanjako et al. targeted, they respectively reported a 97.7% and 85.0% acceptance of HIV testing in the ED [17, 19, 20]. A study in rape survivors by Ranney et al. reported testing acceptance at 88.7%, the lowest testing acceptance was reported by Sawe et al. who conducted a study in pediatric patient and reported a testing acceptance of only 29.5% [21, 29]. Linkage to care feasibility was assessed by Waxman et al. who reported that 85% of newly diagnosed HIV positive patients, compliant with their initial HIV clinic visits and 65% complaint with an additional 1-month follow-up visit [20].

Three studies conduct patient surveys to evaluate the acceptance of an ED-based HIV testing strategy (Table 3). Nakanjako et al. interviewed 233 patients in the ED and reported that 99% of patients support HIV testing in the ED and 86% believed that ED-based testing would improve linkage to care [19]. Christensen el al. sought to determine the acceptability of ED based testing using a closed-question survey [27]. Out of the 343 patients interviewed (using a convenience sample of patients who presented for care in the ED during the study), 75% were found to be amenable to opt-out testing if offered in the ED [27]. Patients greater then 50 years old, females, and those who had not been previously tested were more likely to refuse hypothetical HIV testing [27]. Potential reasons for declining testing were also evaluated. The two most common reasons for declining were, “I have had an HIV test recently enough” (85%, 95% CI 74.0–91.4%) and “I am not at risk for HIV/AIDS” (83%, 95% CI 73.0–90.4%) [27]. Additionally, Christensen and colleagues found that over 30% of patients in their study had never been tested for HIV, with 40% reporting that the ED was their only access to health care [27, 30]. Fear and stigma also played a role in refusal. People cited embarrassment (19%, 11.7–30.4%), rejection (30%, 20.3–41.5%), and being afraid (21%, 12.7–31.8%) as reasons they would decline hypothetical HIV testing in the ED [27].

**Table 3:** A summary of qualitative studies evaluating ED-based HIV testing acceptance.

Country	Year	Journal	Author	Type of study	Description
Guyana	2012	Int. Health	Christensen [27]	Survey	Four-part survey administered to 343 non-critical adult patients
Uganda	2007	AIDS Behav	Nakanako [19]	Survey	A convenience sample of 245 patients were screened of which 233 adults were interviewed and offered HIV testing, data was collected on reasons of prior HIV testing, acceptance to take a test and their current HIV sero-status.
Kenya	2008	AIDS Patient Care and STDs	Waxman [28]	Survey	Descriptive study of staff experience regarding the implementation of an ED-based HIV testing program.

Only a single paper which evaluated the cost implications of ED based testing strategies in our review. Due to the readily available provision of low cost tests, Minz et al. found that testing costs in LMICs are likely to be significantly lower than in high income settings [24].

## Discussion

The HIV epidemic remains a significant contributor to the burden of disease in low resource settings. In the US, ED-based HIV testing strategies have been front and center at curbing the HIV epidemic [31]. This systematic review yielded a surprisingly low number of studies focused on ED-based HIV testing in LMICs (in particular there were no studies from South America or South-East Asia). This may represent the fact that emergency medicine is a relatively new specialty, and in many countries (particularly in LMICs) yet to be formally recognized [32]. In nascent healthcare systems, EDs are often a small, underfunded, and under resourced components of the healthcare delivery model [33]. The limited number of studies we observed may represent the paucity of HIV testing in this venue in LMICs. EDs in LMICs also report significantly higher morbidity and mortality compared to their high-income country counterparts [34]. Even though many LMICs recommend routine HIV testing in all healthcare facilities it may be difficult to implement and sustain testing given the already high burden of healthcare needs of patients in the ED [28, 35, 36]. The lack of ED-based testing studies thus appears to be representative of an overall absence of ED-based testing programs in LMICs where they are most desperately needed.

It was difficult to compare HIV prevalence across studies. Two of the studies (Minz et al. 2010 and Teja et al. 2004), based ED HIV prevalence estimates on patients that had a routine HIV testing done prior to emergent surgery [22, 25]. Other studies based their decision to offer ED based testing on whether patients were symptomatic [20, 23, 24]. Both of these proxies likely mis-represent the true prevalence of HIV infection in ED. The one study that used a unbiased approach, sampled every 6th patient and reported the highest prevalence of HIV infection at 50% [19].

A variety of testing strategies were identified in this review. The advent of inexpensive rapid point of care HIV tests in the mid-1990s shifted the HIV testing paradigm and allowed for decentralized testing without sophisticated laboratory equipment [37]. Traditional laboratory based testing using ELISA only to detect HIV antibodies were challenged by long laboratory delays. Thus, it is not surprising that the majority of studies in this review, utilized rapid point of care tests to offer readily available testing in the ED [17, 19, 20, 25, 26]. Rapid tests on average require 10–20 minutes to complete, with reactive results permitting patient counseling during the ED visit. Unfortunately, they usually require sequential confirmatory testing with ELISA or PCR which can be time consuming. While point of care HIV testing allows for mobility the overall process is more time consuming and labor intensive for the ED staff.

Fourth generation laboratory based ‘combo’ tests were introduced in the late 1990s and include both an ELISA, which tests for HIV antibodies, and the p24 antigen. The advantage to adding the p24 antigen is the ability for near real-time detection of HIV infection before antibodies are produced thus, improving the detection of acute infections that were previously missed during the window period (i.e., a period when patients test negative (by antibody) despite being infected. Recent advances in diagnostics have shortened assay turnaround time to approximately 20 minutes, such that tests can now be added to routinely sent laboratory testing from the ED, and permit high volume and high throughput testing without additional ED labor time and costs. Rapid lab-based assays also lend themselves to an opt-out consent approach (i.e., testing without pre-and post-test counseling, reflexed onto routine laboratory blood tests). While integrated laboratory based testing in the US has already been shown to yield higher testing volumes (given that the majority of ED patients receive blood draws), this may not apply to resource-limited settings, where laboratory service may be unreliable or underutilized due to cost and resource constraints.

In the low resource settings, there is often a paucity of health care workers to provide testing in the ED. An innovative solution in the ED may be the provision of self-testing [38, 39]. Many patient in the ED present with stable injuries, in these cases patients are likely to have long wait times, and are unlikely to receive a blood draw. Shifting the self-testing innovation from community based settings to the ED, may provide an elegant solution to providing 24 hours testing availability in settings where there are not enough providers perform rapid tests and blood based tests are not possible due to delays in getting results. The concern however is that while self-testing has shown high acceptability it may be associated with low rates of linkage to care [40, 41].

Successful linkage to care from the ED was demonstrated by Waxman et al [28]. Timely linkage to care is even more critical in the current HIV treatment climate which advocates changing the threshold of initiating ARV therapy (from a CD4 count <350 cells/mm^3^ to a CD4 count >500 cells/mm^3^) [42]. While the findings from the Waxman et al. study are encouraging, they are equally surprising given that most LMICs EDs are often located in centralized tertiary care hospitals and see patients from a wide catchment area [34]. It is unclear how linkage to care can be sustainably maintained from a transient care facility such as the ED and future work should focus on the feasibility and efficacy of ARV initiation in the ED and the impact of such a strategy both on delivery of sustainable treatment and development of antiviral resistance within the community.

Given the high burden of HIV infection in many of these settings, coupled with the high patient turnover in the ED, the introduction of HIV testing into EDs has serious financial implications on the institution. Despite national recommendations from the CDC in 2006 mandating universal HIV testing, many EDs in the US have not implemented a testing strategy at their institution [43]. In the US, institutions must foot the bill for HIV testing and are unlikely to see the downstream benefits of morbidity and mortality avoided by early treatment initiation. Many healthcare systems in LMICs are government funded, and thus the inducement of population level benefits may allow for the provision for resources required to successfully implement testing in these settings.

### Limitations

The studies presented in this review vary great by scope and sampling strategy. No studies tested all patients, and thus samples were likely not representative of the true ED population, limiting the ability to have a more complete understanding of the true burden of disease. Most studies were performed at single hospitals, and may lack external validity. It is possible that due to limited resources, patients with histories, symptoms, or signs suggestive of HIV were prioritized in the testing algorithms, artificially boosting prevalence findings. The testing strategy also varied greatly among studies and some did not require confirmatory testing as is standard in many developed countries. Nonetheless, despite this variability, these diverse few studies did show a relatively high burden of HIV infection.

## Conclusions

Although the numbers of published reports are few, these diverse studies imply that in HIV endemic low resource settings, EDs carry a large burden of undiagnosed HIV infection. The ED is a strategic venue for targeting testing in low resource settings for early HIV diagnosis. ED-based testing has been shown to be both feasible and acceptable within this environment. The true burden of HIV infection in the ED and the unique demographic features of the HIV positive ED population still remain largely unknown given the lack of a blanket testing strategy. Further research is required on the implementation and cost effectiveness of ED-based testing strategies given the resource constraints and high prevalence of HIV infection in LMICs.

## Supporting information

S1 Appendix Search strategy. (DOCX)

S1 File PRISMA 2009 checklist. (DOC)
