# Supplementary material for: A systematic review of emergency department based HIV testing and linkage to care initiatives in low resource settings
Source: PLoS One. 2017 Nov 2;12(11):e0187443. doi: 10.1371/journal.pone.0187443 (PMC5667894; doi:10.1371/journal.pone.0187443)
Supplement: S1 Appendix — (DOCX) [file pone.0187443.s001.docx]

**Appendix 1**

Pubmed search strategy:

("Emergency Service, Hospital"[mh] OR "Emergency Medicine"[mh] OR "Emergency medicine"[TW] OR "Emergency services"[TW] OR "Emergency department"[TW] OR "Emergency service"[TW] OR "Emergency departments"[TW] OR "ER"[TW] OR "ED"[tw] OR "Emergency room"[TW] OR "Emergency rooms"[TW] OR "Emergency ward"[TW] OR "Emergency Unit"[TW] OR "Trauma Centers"[mh] OR "Trauma Center"[TW] OR "Trauma Centers"[TW] OR “emergency health service”[tw] OR “emergency health services”[tw] OR “emergency medical services”[tw] OR “emergency medical service”[tw] OR "accident and emergency"[tw] OR "accident & emergency"[tw] OR "a&e"[tw] OR "A & E"[tw] OR "acute care" OR "walk in" OR "urgent care")

AND

("HIV"[mh] OR "HIV-1"[mh] OR "HIV-2"[mh] OR "HIV"[tw] OR "HIV-1"[tw] OR "HIV-2"[tw] OR "human immunodeficiency virus"[tw] OR "human immunodeficiency virus 1"[tw] OR "human immunodeficiency virus 2"[tw] OR "human immunodeficiency viruses"[tw] OR "HTLV-III"[tw] OR "human T cell lymphotrophic virus type III"[tw] OR "human T lymphotropic Virus type III"[tw] OR "human t-cell leukemia virus type III"[tw] OR "human t cell leukemia virus type III"[tw] OR "human t-cell lymphotropic virus type III"[tw] OR "human t-lymphotropic virus type III"[tw] OR "LAV-HTLV-III"[tw] OR "lymphadenopathy-associated virus"[tw] OR "lymphadenopathy associated virus"[tw] OR "lymphadenopathy-associated viruses"[tw] OR "lymphadenopathy associated virus"[tw] OR "Acquired Immunodeficiency Syndrome"[mh] OR "acquired immunodeficiency syndrome"[tw] OR "AIDS"[tw] OR "acquired immunologic deficiency syndrome"[tw] OR "acquired immune deficiency syndrome"[tw] OR "acquired immuno-deficiency syndrome"[tw] OR "acquired immuno deficiency syndrome"[tw] OR "acquired immuno-deficiency syndromes"[tw] OR "acquired immuno deficiency syndromes"[tw] OR "acquired immunodeficiency syndromes"[tw])

AND

("developing country"[tiab] OR "developing countries"[tiab] OR "developing nation"[tiab] OR "developing nations"[tiab] OR "developing population"[tiab] OR "developing populations"[tiab] OR "developing world"[tiab] OR "less developed country"[tiab] OR "less developed countries"[tiab] OR "less developed nation"[tiab] OR "less developed nations"[tiab] OR "less developed population"[tiab] OR "less developed populations"[tiab] OR "less developed world"[tiab] OR "lesser developed country"[tiab] OR "lesser developed countries"[tiab] OR "lesser developed nation"[tiab] OR "lesser developed nations"[tiab] OR "lesser developed population"[tiab] OR "lesser developed populations"[tiab] OR "lesser developed world"[tiab] OR "under developed country"[tiab] OR "under developed countries"[tiab] OR "under developed nation"[tiab] OR "under developed nations"[tiab] OR "under developed population"[tiab] OR "under developed populations"[tiab] OR "under developed world"[tiab] OR "underdeveloped country"[tiab] OR "underdeveloped countries"[tiab] OR "underdeveloped nation"[tiab] OR "underdeveloped nations"[tiab] OR "underdeveloped population"[tiab] OR "underdeveloped populations"[tiab] OR "underdeveloped world"[tiab] OR "middle income country"[tiab] OR "middle income countries"[tiab] OR "middle income nation"[tiab] OR "middle income nations"[tiab] OR "middle income population"[tiab] OR "middle income populations"[tiab] OR "low income country"[tiab] OR "low income countries"[tiab] OR "low income nation"[tiab] OR "low income nations"[tiab] OR "low income population"[tiab] OR "low income populations"[tiab] OR "lower income country"[tiab] OR "lower income countries"[tiab] OR "lower income nation"[tiab] OR "lower income nations"[tiab] OR "lower income population"[tiab] OR "lower income populations"[tiab] OR "underserved country"[tiab] OR "underserved countries"[tiab] OR "underserved nation"[tiab] OR "underserved nations"[tiab] OR "underserved population"[tiab] OR "underserved populations"[tiab] OR "underserved world"[tiab] OR "under served country"[tiab] OR "under served countries"[tiab] OR "under served nation"[tiab] OR "under served nations"[tiab] OR "under served population"[tiab] OR "under served populations"[tiab] OR "under served world"[tiab] OR "deprived country"[tiab] OR "deprived countries"[tiab] OR "deprived nation"[tiab] OR "deprived nations"[tiab] OR "deprived population"[tiab] OR "deprived populations"[tiab] OR "deprived world"[tiab] OR "poor country"[tiab] OR "poor countries"[tiab] OR "poor nation"[tiab] OR "poor nations"[tiab] OR "poor population"[tiab] OR "poor populations"[tiab] OR "poor world"[tiab] OR "poorer country"[tiab] OR "poorer countries"[tiab] OR "poorer nation"[tiab] OR "poorer nations"[tiab] OR "poorer population"[tiab] OR "poorer populations"[tiab] OR "poorer world"[tiab] OR "developing economy"[tiab] OR "developing economies"[tiab] OR "less developed economy"[tiab] OR "less developed economies"[tiab] OR "lesser developed economy"[tiab] OR "lesser developed economies"[tiab] OR "under developed economy"[tiab] OR "under developed economies"[tiab] OR "underdeveloped economy"[tiab] OR "underdeveloped economies"[tiab] OR "middle income economy"[tiab] OR "middle income economies"[tiab] OR "low income economy"[tiab] OR "low income economies"[tiab] OR "lower income economy"[tiab] OR "lower income economies"[tiab] OR "low gdp"[tiab] OR "low gnp"[tiab] OR "low gross domestic"[tiab] OR "low gross national"[tiab] OR "lower gdp"[tiab] OR "lower gnp"[tiab] OR "lower gross domestic"[tiab] OR "lower gross national"[tiab] OR lmic[tiab] OR lmics[tiab] OR "third world"[tiab] OR "lami country"[tiab] OR "lami countries"[tiab] OR "transitional country"[tiab] OR "transitional countries"[tiab] OR "developing country"[ot] OR "developing countries"[ot] OR "developing nation"[ot] OR "developing nations"[ot] OR "developing population"[ot] OR "developing populations"[ot] OR "developing world"[ot] OR "less developed country"[ot] OR "less developed countries"[ot] OR "less developed nation"[ot] OR "less developed nations"[ot] OR "less developed population"[ot] OR "less developed populations"[ot] OR "less developed world"[ot] OR "lesser developed country"[ot] OR "lesser developed countries"[ot] OR "lesser developed nation"[ot] OR "lesser developed nations"[ot] OR "lesser developed population"[ot] OR "lesser developed populations"[ot] OR "lesser developed world"[ot] OR "under developed country"[ot] OR "under developed countries"[ot] OR "under developed nation"[ot] OR "under developed nations"[ot] OR "under developed population"[ot] OR "under developed populations"[ot] OR "under developed world"[ot] OR "underdeveloped country"[ot] OR "underdeveloped countries"[ot] OR "underdeveloped nation"[ot] OR "underdeveloped nations"[ot] OR "underdeveloped population"[ot] OR "underdeveloped populations"[ot] OR "underdeveloped world"[ot] OR "middle income country"[ot] OR "middle income countries"[ot] OR "middle income nation"[ot] OR "middle income nations"[ot] OR "middle income population"[ot] OR "middle income populations"[ot] OR "low income country"[ot] OR "low income countries"[ot] OR "low income nation"[ot] OR "low income nations"[ot] OR "low income population"[ot] OR "low income populations"[ot] OR "lower income country"[ot] OR "lower income countries"[ot] OR "lower income nation"[ot] OR "lower income nations"[ot] OR "lower income population"[ot] OR "lower income populations"[ot] OR "underserved country"[ot] OR "underserved countries"[ot] OR "underserved nation"[ot] OR "underserved nations"[ot] OR "underserved population"[ot] OR "underserved populations"[ot] OR "underserved world"[ot] OR "under served country"[ot] OR "under served countries"[ot] OR "under served nation"[ot] OR "under served nations"[ot] OR "under served population"[ot] OR "under served populations"[ot] OR "under served world"[ot] OR "deprived country"[ot] OR "deprived countries"[ot] OR "deprived nation"[ot] OR "deprived nations"[ot] OR "deprived population"[ot] OR "deprived populations"[ot] OR "deprived world"[ot] OR "poor country"[ot] OR "poor countries"[ot] OR "poor nation"[ot] OR "poor nations"[ot] OR "poor population"[ot] OR "poor populations"[ot] OR "poor world"[ot] OR "poorer country"[ot] OR "poorer countries"[ot] OR "poorer nation"[ot] OR "poorer nations"[ot] OR "poorer population"[ot] OR "poorer populations"[ot] OR "poorer world"[ot] OR "developing economy"[ot] OR "developing economies"[ot] OR "less developed economy"[ot] OR "less developed economies"[ot] OR "lesser developed economy"[ot] OR "lesser developed economies"[ot] OR "under developed economy"[ot] OR "under developed economies"[ot] OR "underdeveloped economy"[ot] OR "underdeveloped economies"[ot] OR "middle income economy"[ot] OR "middle income economies"[ot] OR "low income economy"[ot] OR "low income economies"[ot] OR "lower income economy"[ot] OR "lower income economies"[ot] OR "low gdp"[ot] OR "low gnp"[ot] OR "low gross domestic"[ot] OR "low gross national"[ot] OR "lower gdp"[ot] OR "lower gnp"[ot] OR "lower gross domestic"[ot] OR "lower gross national"[ot] OR lmic[ot] OR lmics[ot] OR "third world"[ot] OR "lami country"[ot] OR "lami countries"[ot] OR "transitional country"[ot] OR "transitional countries"[ot] OR Africa[pl] OR Asia[pl] OR Caribbean[pl] OR West Indies[pl] OR South America[pl] OR Latin America[pl] OR Central America[pl] OR Afghanistan[pl] OR Albania[pl] OR Algeria[pl] OR Angola[pl] OR Antigua[pl] OR Barbuda[pl] OR Argentina[pl] OR Armenia[pl] OR Armenian[pl] OR Aruba[pl] OR Azerbaijan[pl] OR Bahrain[pl] OR Bangladesh[pl] OR Barbados[pl] OR Benin[pl] OR Byelarus[pl] OR Byelorussian[pl] OR Belarus[pl] OR Belorussian[pl] OR Belorussia[pl] OR Belize[pl] OR Bhutan[pl] OR Bolivia[pl] OR Bosnia[pl] OR Herzegovina[pl] OR Hercegovina[pl] OR Botswana[pl] OR Brasil[pl] OR Brazil[pl] OR Bulgaria[pl] OR Burkina Faso[pl] OR Burkina Fasso[pl] OR Upper Volta[pl] OR Burundi[pl] OR Urundi[pl] OR Cambodia[pl] OR Khmer Republic[pl] OR Kampuchea[pl] OR Cameroon[pl] OR Cameroons[pl] OR Cameron[pl] OR Camerons[pl] OR Cape Verde[pl] OR Central African Republic[pl] OR Chad[pl] OR Chile[pl] OR China[pl] OR Colombia[pl] OR Comoros[pl] OR Comoro Islands[pl] OR Comores[pl] OR Mayotte[pl] OR Congo[pl] OR Zaire[pl] OR Costa Rica[pl] OR Cote d'Ivoire[pl] OR Ivory Coast[pl] OR Croatia[pl] OR Cuba[pl] OR Cyprus[pl] OR Czechoslovakia[pl] OR Czech Republic[pl] OR Slovakia[pl] OR Slovak Republic[pl] OR Djibouti[pl] OR French Somaliland[pl] OR Dominica[pl] OR Dominican Republic[pl] OR East Timor[pl] OR East Timur[pl] OR Timor Leste[pl] OR Ecuador[pl] OR Egypt[pl] OR United Arab Republic[pl] OR El Salvador[pl] OR Eritrea[pl] OR Estonia[pl] OR Ethiopia[pl] OR Fiji[pl] OR Gabon[pl] OR Gabonese Republic[pl] OR Gambia[pl] OR Gaza[pl] OR Georgia Republic[pl] OR Georgian Republic[pl] OR Ghana[pl] OR Gold Coast[pl] OR Greece[pl] OR Grenada[pl] OR Guatemala[pl] OR Guinea[pl] OR Guam[pl] OR Guiana[pl] OR Guyana[pl] OR Haiti[pl] OR Honduras[pl] OR Hungary[pl] OR India[pl] OR Maldives[pl] OR Indonesia[pl] OR Iran[pl] OR Iraq[pl] OR Isle of Man[pl] OR Jamaica[pl] OR Jordan[pl] OR Kazakhstan[pl] OR Kazakh[pl] OR Kenya[pl] OR Kiribati[pl] OR Korea[pl] OR Kosovo[pl] OR Kyrgyzstan[pl] OR Kirghizia[pl] OR Kyrgyz Republic[pl] OR Kirghiz[pl] OR Kirgizstan[pl] OR "Lao PDR"[pl] OR Laos[pl] OR Latvia[pl] OR Lebanon[pl] OR Lesotho[pl] OR Basutoland[pl] OR Liberia[pl] OR Libya[pl] OR Lithuania[pl] OR Macedonia[pl] OR Madagascar[pl] OR Malagasy Republic[pl] OR Malaysia[pl] OR Malaya[pl] OR Malay[pl] OR Sabah[pl] OR Sarawak[pl] OR Malawi[pl] OR Nyasaland[pl] OR Mali[pl] OR Malta[pl] OR Marshall Islands[pl] OR Mauritania[pl] OR Mauritius[pl] OR Agalega Islands[pl] OR Mexico[pl] OR Micronesia[pl] OR Middle East[pl] OR Moldova[pl] OR Moldovia[pl] OR Moldovian[pl] OR Mongolia[pl] OR Montenegro[pl] OR Morocco[pl] OR Ifni[pl] OR Mozambique[pl] OR Myanmar[pl] OR Myanma[pl] OR Burma[pl] OR Namibia[pl] OR Nepal[pl] OR Netherlands Antilles[pl] OR New Caledonia[pl] OR Nicaragua[pl] OR Niger[pl] OR Nigeria[pl] OR Northern Mariana Islands[pl] OR Oman[pl] OR Muscat[pl] OR Pakistan[pl] OR Palau[pl] OR Palestine[pl] OR Panama[pl] OR Paraguay[pl] OR Peru[pl] OR Philippines[pl] OR Philipines[pl] OR Phillipines[pl] OR Phillippines[pl] OR Poland[pl] OR Portugal[pl] OR Puerto Rico[pl] OR Romania[pl] OR Rumania[pl] OR Roumania[pl] OR Russia[pl] OR Russian[pl] OR Rwanda[pl] OR Ruanda[pl] OR Saint Kitts[pl] OR St Kitts[pl] OR Nevis[pl] OR Saint Lucia[pl] OR St Lucia[pl] OR Saint Vincent[pl] OR St Vincent[pl] OR Grenadines[pl] OR Samoa[pl] OR Samoan Islands[pl] OR Navigator Island[pl] OR Navigator Islands[pl] OR Sao Tome[pl] OR Saudi Arabia[pl] OR Senegal[pl] OR Serbia[pl] OR Montenegro[pl] OR Seychelles[pl] OR Sierra Leone[pl] OR Slovenia[pl] OR Sri Lanka[pl] OR Ceylon[pl] OR Solomon Islands[pl] OR Somalia[pl] OR South Africa[pl] OR Sudan[pl] OR Suriname[pl] OR Surinam[pl] OR Swaziland[pl] OR Syria[pl] OR Tajikistan[pl] OR Tadzhikistan[pl] OR Tadjikistan[pl] OR Tadzhik[pl] OR Tanzania[pl] OR Thailand[pl] OR Togo[pl] OR Togolese Republic[pl] OR Tonga[pl] OR Trinidad[pl] OR Tobago[pl] OR Tunisia[pl] OR Turkey[pl] OR Turkmenistan[pl] OR Turkmen[pl] OR Uganda[pl] OR Ukraine[pl] OR Uruguay[pl] OR USSR[pl] OR Soviet Union[pl] OR Union of Soviet Socialist Republics[pl] OR Uzbekistan[pl] OR Uzbek OR Vanuatu[pl] OR New Hebrides[pl] OR Venezuela[pl] OR Vietnam[pl] OR Viet Nam[pl] OR West Bank[pl] OR Yemen[pl] OR Yugoslavia[pl] OR Zambia[pl] OR Zimbabwe[pl] OR Rhodesia[pl] OR Africa[tiab] OR Asia[tiab] OR Caribbean[tiab] OR West Indies[tiab] OR South America[tiab] OR Latin America[tiab] OR Central America[tiab] OR Afghanistan[tiab] OR Albania[tiab] OR Algeria[tiab] OR Angola[tiab] OR Antigua[tiab] OR Barbuda[tiab] OR Argentina[tiab] OR Armenia[tiab] OR Armenian[tiab] OR Aruba[tiab] OR Azerbaijan[tiab] OR Bahrain[tiab] OR Bangladesh[tiab] OR Barbados[tiab] OR Benin[tiab] OR Byelarus[tiab] OR Byelorussian[tiab] OR Belarus[tiab] OR Belorussian[tiab] OR Belorussia[tiab] OR Belize[tiab] OR Bhutan[tiab] OR Bolivia[tiab] OR Bosnia[tiab] OR Herzegovina[tiab] OR Hercegovina[tiab] OR Botswana[tiab] OR Brasil[tiab] OR Brazil[tiab] OR Bulgaria[tiab] OR Burkina Faso[tiab] OR Burkina Fasso[tiab] OR Upper Volta[tiab] OR Burundi[tiab] OR Urundi[tiab] OR Cambodia[tiab] OR Khmer Republic[tiab] OR Kampuchea[tiab] OR Cameroon[tiab] OR Cameroons[tiab] OR Cameron[tiab] OR Camerons[tiab] OR Cape Verde[tiab] OR Central African Republic[tiab] OR Chad[tiab] OR Chile[tiab] OR China[tiab] OR Colombia[tiab] OR Comoros[tiab] OR Comoro Islands[tiab] OR Comores[tiab] OR Mayotte[tiab] OR Congo[tiab] OR Zaire[tiab] OR Costa Rica[tiab] OR Cote d'Ivoire[tiab] OR Ivory Coast[tiab] OR Croatia[tiab] OR Cuba[tiab] OR Cyprus[tiab] OR Czechoslovakia[tiab] OR Czech Republic[tiab] OR Slovakia[tiab] OR Slovak Republic[tiab] OR Djibouti[tiab] OR French Somaliland[tiab] OR Dominica[tiab] OR Dominican Republic[tiab] OR East Timor[tiab] OR East Timur[tiab] OR Timor Leste[tiab] OR Ecuador[tiab] OR Egypt[tiab] OR United Arab Republic[tiab] OR El Salvador[tiab] OR Eritrea[tiab] OR Estonia[tiab] OR Ethiopia[tiab] OR Fiji[tiab] OR Gabon[tiab] OR Gabonese Republic[tiab] OR Gambia[tiab] OR Gaza[tiab] OR Georgia Republic[tiab] OR Georgian Republic[tiab] OR Ghana[tiab] OR Gold Coast[tiab] OR Greece[tiab] OR Grenada[tiab] OR Guatemala[tiab] OR Guinea[tiab] OR Guam[tiab] OR Guiana[tiab] OR Guyana[tiab] OR Haiti[tiab] OR Honduras[tiab] OR Hungary[tiab] OR India[tiab] OR Maldives[tiab] OR Indonesia[tiab] OR Iran[tiab] OR Iraq[tiab] OR Isle of Man[tiab] OR Jamaica[tiab] OR Jordan[tiab] OR Kazakhstan[tiab] OR Kazakh[tiab] OR Kenya[tiab] OR Kiribati[tiab] OR Korea[tiab] OR Kosovo[tiab] OR Kyrgyzstan[tiab] OR Kirghizia[tiab] OR Kyrgyz Republic[tiab] OR Kirghiz[tiab] OR Kirgizstan[tiab] OR "Lao PDR"[tiab] OR Laos[tiab] OR Latvia[tiab] OR Lebanon[tiab] OR Lesotho[tiab] OR Basutoland[tiab] OR Liberia[tiab] OR Libya[tiab] OR Lithuania[tiab] OR Macedonia[tiab] OR Madagascar[tiab] OR Malagasy Republic[tiab] OR Malaysia[tiab] OR Malaya[tiab] OR Malay[tiab] OR Sabah[tiab] OR Sarawak[tiab] OR Malawi[tiab] OR Nyasaland[tiab] OR Mali[tiab] OR Malta[tiab] OR Marshall Islands[tiab] OR Mauritania[tiab] OR Mauritius[tiab] OR Agalega Islands[tiab] OR Mexico[tiab] OR Micronesia[tiab] OR Middle East[tiab] OR Moldova[tiab] OR Moldovia[tiab] OR Moldovian[tiab] OR Mongolia[tiab] OR Montenegro[tiab] OR Morocco[tiab] OR Ifni[tiab] OR Mozambique[tiab] OR Myanmar[tiab] OR Myanma[tiab] OR Burma[tiab] OR Namibia[tiab] OR Nepal[tiab] OR Netherlands Antilles[tiab] OR New Caledonia[tiab] OR Nicaragua[tiab] OR Niger[tiab] OR Nigeria[tiab] OR Northern Mariana Islands[tiab] OR Oman[tiab] OR Muscat[tiab] OR Pakistan[tiab] OR Palau[tiab] OR Palestine[tiab] OR Panama[tiab] OR Paraguay[tiab] OR Peru[tiab] OR Philippines[tiab] OR Philipines[tiab] OR Phillipines[tiab] OR Phillippines[tiab] OR Poland[tiab] OR Portugal[tiab] OR Puerto Rico[tiab] OR Romania[tiab] OR Rumania[tiab] OR Roumania[tiab] OR Russia[tiab] OR Russian[tiab] OR Rwanda[tiab] OR Ruanda[tiab] OR Saint Kitts[tiab] OR St Kitts[tiab] OR Nevis[tiab] OR Saint Lucia[tiab] OR St Lucia[tiab] OR Saint Vincent[tiab] OR St Vincent[tiab] OR Grenadines[tiab] OR Samoa[tiab] OR Samoan Islands[tiab] OR Navigator Island[tiab] OR Navigator Islands[tiab] OR Sao Tome[tiab] OR Saudi Arabia[tiab] OR Senegal[tiab] OR Serbia[tiab] OR Montenegro[tiab] OR Seychelles[tiab] OR Sierra Leone[tiab] OR Slovenia[tiab] OR Sri Lanka[tiab] OR Ceylon[tiab] OR Solomon Islands[tiab] OR Somalia[tiab] OR Sudan[tiab] OR Suriname[tiab] OR Surinam[tiab] OR Swaziland[tiab] OR Syria[tiab] OR Tajikistan[tiab] OR Tadzhikistan[tiab] OR Tadjikistan[tiab] OR Tadzhik[tiab] OR Tanzania[tiab] OR Thailand[tiab] OR Togo[tiab] OR Togolese Republic[tiab] OR Tonga[tiab] OR Trinidad[tiab] OR Tobago[tiab] OR Tunisia[tiab] OR Turkey[tiab] OR Turkmenistan[tiab] OR Turkmen[tiab] OR Uganda[tiab] OR Ukraine[tiab] OR Uruguay[tiab] OR USSR[tiab] OR Soviet Union[tiab] OR Union of Soviet Socialist Republics[tiab] OR Uzbekistan[tiab] OR Uzbek OR Vanuatu[tiab] OR New Hebrides[tiab] OR Venezuela[tiab] OR Vietnam[tiab] OR Viet Nam[tiab] OR West Bank[tiab] OR Yemen[tiab] OR Yugoslavia[tiab] OR Zambia[tiab] OR Zimbabwe[tiab] OR Rhodesia[tiab] OR Africa[ot] OR Asia[ot] OR Caribbean[ot] OR West Indies[ot] OR South America[ot] OR Latin America[ot] OR Central America[ot] OR Afghanistan[ot] OR Albania[ot] OR Algeria[ot] OR Angola[ot] OR Antigua[ot] OR Barbuda[ot] OR Argentina[ot] OR Armenia[ot] OR Armenian[ot] OR Aruba[ot] OR Azerbaijan[ot] OR Bahrain[ot] OR Bangladesh[ot] OR Barbados[ot] OR Benin[ot] OR Byelarus[ot] OR Byelorussian[ot] OR Belarus[ot] OR Belorussian[ot] OR Belorussia[ot] OR Belize[ot] OR Bhutan[ot] OR Bolivia[ot] OR Bosnia[ot] OR Herzegovina[ot] OR Hercegovina[ot] OR Botswana[ot] OR Brasil[ot] OR Brazil[ot] OR Bulgaria[ot] OR Burkina Faso[ot] OR Burkina Fasso[ot] OR Upper Volta[ot] OR Burundi[ot] OR Urundi[ot] OR Cambodia[ot] OR Khmer Republic[ot] OR Kampuchea[ot] OR Cameroon[ot] OR Cameroons[ot] OR Cameron[ot] OR Camerons[ot] OR Cape Verde[ot] OR Central African Republic[ot] OR Chad[ot] OR Chile[ot] OR China[ot] OR Colombia[ot] OR Comoros[ot] OR Comoro Islands[ot] OR Comores[ot] OR Mayotte[ot] OR Congo[ot] OR Zaire[ot] OR Costa Rica[ot] OR Cote d'Ivoire[ot] OR Ivory Coast[ot] OR Croatia[ot] OR Cuba[ot] OR Cyprus[ot] OR Czechoslovakia[ot] OR Czech Republic[ot] OR Slovakia[ot] OR Slovak Republic[ot] OR Djibouti[ot] OR French Somaliland[ot] OR Dominica[ot] OR Dominican Republic[ot] OR East Timor[ot] OR East Timur[ot] OR Timor Leste[ot] OR Ecuador[ot] OR Egypt[ot] OR United Arab Republic[ot] OR El Salvador[ot] OR Eritrea[ot] OR Estonia[ot] OR Ethiopia[ot] OR Fiji[ot] OR Gabon[ot] OR Gabonese Republic[ot] OR Gambia[ot] OR Gaza[ot] OR "Georgia Republic"[ot] OR "Georgian Republic"[ot] OR Ghana[ot] OR Gold Coast[ot] OR Greece[ot] OR Grenada[ot] OR Guatemala[ot] OR Guinea[ot] OR Guam[ot] OR Guiana[ot] OR Guyana[ot] OR Haiti[ot] OR Honduras[ot] OR Hungary[ot] OR India[ot] OR Maldives[ot] OR Indonesia[ot] OR Iran[ot] OR Iraq[ot] OR Isle of Man[ot] OR Jamaica[ot] OR Jordan[ot] OR Kazakhstan[ot] OR Kazakh[ot] OR Kenya[ot] OR Kiribati[ot] OR Korea[ot] OR Kosovo[ot] OR Kyrgyzstan[ot] OR Kirghizia[ot] OR Kyrgyz Republic[ot] OR Kirghiz[ot] OR Kirgizstan[ot] OR "Lao PDR"[ot] OR Laos[ot] OR Latvia[ot] OR Lebanon[ot] OR Lesotho[ot] OR Basutoland[ot] OR Liberia[ot] OR Libya[ot] OR Lithuania[ot] OR Macedonia[ot] OR Madagascar[ot] OR Malagasy Republic[ot] OR Malaysia[ot] OR Malaya[ot] OR Malay[ot] OR Sabah[ot] OR Sarawak[ot] OR Malawi[ot] OR Nyasaland[ot] OR Mali[ot] OR Malta[ot] OR Marshall Islands[ot] OR Mauritania[ot] OR Mauritius[ot] OR Agalega Islands[ot] OR Mexico[ot] OR Micronesia[ot] OR Middle East[ot] OR Moldova[ot] OR Moldovia[ot] OR Moldovian[ot] OR Mongolia[ot] OR Montenegro[ot] OR Morocco[ot] OR Ifni[ot] OR Mozambique[ot] OR Myanmar[ot] OR Myanma[ot] OR Burma[ot] OR Namibia[ot] OR Nepal[ot] OR Netherlands Antilles[ot] OR New Caledonia[ot] OR Nicaragua[ot] OR Niger[ot] OR Nigeria[ot] OR Northern Mariana Islands[ot] OR Oman[ot] OR Muscat[ot] OR Pakistan[ot] OR Palau[ot] OR Palestine[ot] OR Panama[ot] OR Paraguay[ot] OR Peru[ot] OR Philippines[ot] OR Philipines[ot] OR Phillipines[ot] OR Phillippines[ot] OR Poland[ot] OR Portugal[ot] OR Puerto Rico[ot] OR Romania[ot] OR Rumania[ot] OR Roumania[ot] OR Russia[ot] OR Russian[ot] OR Rwanda[ot] OR Ruanda[ot] OR Saint Kitts[ot] OR St Kitts[ot] OR Nevis[ot] OR Saint Lucia[ot] OR St Lucia[ot] OR Saint Vincent[ot] OR St Vincent[ot] OR Grenadines[ot] OR Samoa[ot] OR Samoan Islands[ot] OR Navigator Island[ot] OR Navigator Islands[ot] OR Sao Tome[ot] OR Saudi Arabia[ot] OR Senegal[ot] OR Serbia[ot] OR Montenegro[ot] OR Seychelles[ot] OR Sierra Leone[ot] OR Slovenia[ot] OR Sri Lanka[ot] OR Ceylon[ot] OR Solomon Islands[ot] OR Somalia[ot] OR Sudan[ot] OR Suriname[ot] OR Surinam[ot] OR Swaziland[ot] OR Syria[ot] OR Tajikistan[ot] OR Tadzhikistan[ot] OR Tadjikistan[ot] OR Tadzhik[ot] OR Tanzania[ot] OR Thailand[ot] OR Togo[ot] OR Togolese Republic[ot] OR Tonga[ot] OR Trinidad[ot] OR Tobago[ot] OR Tunisia[ot] OR Turkey[ot] OR Turkmenistan[ot] OR Turkmen[ot] OR Uganda[ot] OR Ukraine[ot] OR Uruguay[ot] OR USSR[ot] OR Soviet Union[ot] OR Union of Soviet Socialist Republics[ot] OR Uzbekistan[ot] OR Uzbek OR Vanuatu[ot] OR New Hebrides[ot] OR Venezuela[ot] OR Vietnam[ot] OR Viet Nam[ot] OR West Bank[ot] OR Yemen[ot] OR Yugoslavia[ot] OR Zambia[ot] OR Zimbabwe[ot] OR Rhodesia[ot] OR Developing Countries[Mesh:noexp] OR Africa[Mesh:noexp] OR Africa, Northern[Mesh:noexp] OR Africa South of the Sahara[Mesh:noexp] OR Africa, Central[Mesh:noexp] OR Africa, Eastern[Mesh:noexp] OR Africa, Southern[Mesh:noexp] OR Africa, Western[Mesh:noexp] OR Asia[Mesh:noexp] OR Asia, Central[Mesh:noexp] OR Asia, Southeastern[Mesh:noexp] OR Asia, Western[Mesh:noexp] OR Caribbean Region[Mesh:noexp] OR West Indies[Mesh:noexp] OR South America[Mesh:noexp] OR Latin America[Mesh:noexp] OR Central America[Mesh:noexp] OR Afghanistan[Mesh:noexp] OR Albania[Mesh:noexp] OR Algeria[Mesh:noexp] OR American Samoa[Mesh:noexp] OR Angola[Mesh:noexp] OR "Antigua and Barbuda"[Mesh:noexp] OR Argentina[Mesh:noexp] OR Armenia[Mesh:noexp] OR Azerbaijan[Mesh:noexp] OR Bahrain[Mesh:noexp] OR Bangladesh[Mesh:noexp] OR Barbados[Mesh:noexp] OR Benin[Mesh:noexp] OR Byelarus[Mesh:noexp] OR Belize[Mesh:noexp] OR Bhutan[Mesh:noexp] OR Bolivia[Mesh:noexp] OR Bosnia-Herzegovina[Mesh:noexp] OR Botswana[Mesh:noexp] OR Brazil[Mesh:noexp] OR Bulgaria[Mesh:noexp] OR Burkina Faso[Mesh:noexp] OR Burundi[Mesh:noexp] OR Cambodia[Mesh:noexp] OR Cameroon[Mesh:noexp] OR Cape Verde[Mesh:noexp] OR Central African Republic[Mesh:noexp] OR Chad[Mesh:noexp] OR Chile[Mesh:noexp] OR China[Mesh:noexp] OR Colombia[Mesh:noexp] OR Comoros[Mesh:noexp] OR Congo[Mesh:noexp] OR Costa Rica[Mesh:noexp] OR Cote d'Ivoire[Mesh:noexp] OR Croatia[Mesh:noexp] OR Cuba[Mesh:noexp] OR Cyprus[Mesh:noexp] OR Czechoslovakia[Mesh:noexp] OR Czech Republic[Mesh:noexp] OR Slovakia[Mesh:noexp] OR Djibouti[Mesh:noexp] OR "Democratic Republic of the Congo"[Mesh:noexp] OR Dominica[Mesh:noexp] OR Dominican Republic[Mesh:noexp] OR East Timor[Mesh:noexp] OR Ecuador[Mesh:noexp] OR Egypt[Mesh:noexp] OR El Salvador[Mesh:noexp] OR Eritrea[Mesh:noexp] OR Estonia[Mesh:noexp] OR Ethiopia[Mesh:noexp] OR Fiji[Mesh:noexp] OR Gabon[Mesh:noexp] OR Gambia[Mesh:noexp] OR "Georgia (Republic)"[Mesh:noexp] OR Ghana[Mesh:noexp] OR Greece[Mesh:noexp] OR Grenada[Mesh:noexp] OR Guatemala[Mesh:noexp] OR Guinea[Mesh:noexp] OR Guinea-Bissau[Mesh:noexp] OR Guam[Mesh:noexp] OR Guyana[Mesh:noexp] OR Haiti[Mesh:noexp] OR Honduras[Mesh:noexp] OR Hungary[Mesh:noexp] OR India[Mesh:noexp] OR Indonesia[Mesh:noexp] OR Iran[Mesh:noexp] OR Iraq[Mesh:noexp] OR Jamaica[Mesh:noexp] OR Jordan[Mesh:noexp] OR Kazakhstan[Mesh:noexp] OR Kenya[Mesh:noexp] OR Korea[Mesh:noexp] OR Kosovo[Mesh:noexp] OR Kyrgyzstan[Mesh:noexp] OR Laos[Mesh:noexp] OR Latvia[Mesh:noexp] OR Lebanon[Mesh:noexp] OR Lesotho[Mesh:noexp] OR Liberia[Mesh:noexp] OR Libya[Mesh:noexp] OR Lithuania[Mesh:noexp] OR Macedonia[Mesh:noexp] OR Madagascar[Mesh:noexp] OR Malaysia[Mesh:noexp] OR Malawi[Mesh:noexp] OR Mali[Mesh:noexp] OR Malta[Mesh:noexp] OR Mauritania[Mesh:noexp] OR Mauritius[Mesh:noexp] OR Mexico[Mesh:noexp] OR Micronesia[Mesh:noexp] OR Middle East[Mesh:noexp] OR Moldova[Mesh:noexp] OR Mongolia[Mesh:noexp] OR Montenegro[Mesh:noexp] OR Morocco[Mesh:noexp] OR Mozambique[Mesh:noexp] OR Myanmar[Mesh:noexp] OR Namibia[Mesh:noexp] OR Nepal[Mesh:noexp] OR Netherlands Antilles[Mesh:noexp] OR New Caledonia[Mesh:noexp] OR Nicaragua[Mesh:noexp] OR Niger[Mesh:noexp] OR Nigeria[Mesh:noexp] OR Oman[Mesh:noexp] OR Pakistan[Mesh:noexp] OR Palau[Mesh:noexp] OR Panama[Mesh:noexp] OR Papua New Guinea[Mesh:noexp] OR Paraguay[Mesh:noexp] OR Peru[Mesh:noexp] OR Philippines[Mesh:noexp] OR Poland[Mesh:noexp] OR Portugal[Mesh:noexp] OR Puerto Rico[Mesh:noexp] OR Romania[Mesh:noexp] OR Russia[Mesh:noexp] OR "Russia (Pre-1917)"[Mesh:noexp] OR Rwanda[Mesh:noexp] OR "Saint Kitts and Nevis"[Mesh:noexp] OR Saint Lucia[Mesh:noexp] OR "Saint Vincent and the Grenadines"[Mesh:noexp] OR Samoa[Mesh:noexp] OR Saudi Arabia[Mesh:noexp] OR Senegal[Mesh:noexp] OR Serbia[Mesh:noexp] OR Montenegro[Mesh:noexp] OR Seychelles[Mesh:noexp] OR Sierra Leone[Mesh:noexp] OR Slovenia[Mesh:noexp] OR Sri Lanka[Mesh:noexp] OR Somalia[Mesh:noexp] OR South Africa[Mesh:noexp] OR Sudan[Mesh:noexp] OR Suriname[Mesh:noexp] OR Swaziland[Mesh:noexp] OR Syria[Mesh:noexp] OR Tajikistan[Mesh:noexp] OR Tanzania[Mesh:noexp] OR Thailand[Mesh:noexp] OR Togo[Mesh:noexp] OR Tonga[Mesh:noexp] OR "Trinidad and Tobago"[Mesh:noexp] OR Tunisia[Mesh:noexp] OR Turkey[Mesh:noexp] OR Turkmenistan[Mesh:noexp] OR Uganda[Mesh:noexp] OR Ukraine[Mesh:noexp] OR Uruguay[Mesh:noexp] OR USSR[Mesh:noexp] OR Uzbekistan[Mesh:noexp] OR Vanuatu[Mesh:noexp] OR Venezuela[Mesh:noexp] OR Vietnam[Mesh:noexp] OR Yemen[Mesh:noexp] OR Yugoslavia[Mesh:noexp] OR Zambia[Mesh:noexp] OR Zimbabwe[Mesh:noexp])

Embase search strategy:

('emergency health service'/exp OR 'emergency medicine'/exp OR 'emergency ward'/exp OR 'emergency health service' OR 'emergency medicine':ab,ti,de,au,lnk OR 'emergency services' OR 'emergency department':ab,ti,de,au,lnk OR 'emergency service' OR 'emergency departments' OR 'ER' OR 'ED' OR 'emergency room' OR 'emergency rooms' OR 'emergency ward' OR 'emergency unit' OR 'trauma centers' OR 'trauma center' OR 'emergency health services' OR 'emergency medical services' OR 'emergency medical service' OR 'accident & emergency' OR 'accident and emergency' OR 'A&E' OR 'A & E')

AND

('HIV' OR 'HIV-1' OR 'HIV-2' OR 'human immunodeficiency virus'/exp OR 'human immunodeficiency virus 1'/exp OR 'human immunodeficiency virus 2'/exp OR 'human immunodeficiency virus' OR 'human immunodeficiency viruses' OR 'HTLV-III' OR 'human T cell lymphotrophic virus type III' OR 'human T lymphotropic Virus type III' OR 'human t-cell leukemia virus type III' OR 'human t cell leukemia virus type III' OR 'human t-cell lymphotropic virus type III' OR 'human t-lymphotropic virus type III' OR 'LAV-HTLV-III' OR 'lymphadenopathy-associated virus' OR 'lymphadenopathy associated virus' OR 'lymphadenopathy-associated viruses' OR 'lymphadenopathy associated virus' OR 'Acquired Immunodeficiency Syndrome' OR 'AIDS' OR 'acquired immunologic deficiency syndrome' OR 'acquired immune deficiency syndrome'/de OR 'acquired immune deficiency syndrome':ti,ab OR 'acquired immuno-deficiency syndrome' OR 'acquired immuno deficiency syndrome' OR 'acquired immuno-deficiency syndromes' OR 'acquired immuno deficiency syndromes' OR 'acquired immunodeficiency syndromes')

AND

**Combine: (#1 OR #2 OR #3 OR #4 OR #5 OR #6 OR #7)**

**#1 Search** 'developing country':ab,ti OR 'developing countries':ab,ti OR 'developing nation':ab,ti OR 'developing nations':ab,ti OR 'developing population':ab,ti OR 'developing populations':ab,ti OR 'developing world':ab,ti OR 'less developed country':ab,ti OR 'less developed countries':ab,ti OR 'less developed nation':ab,ti OR 'less developed nations':ab,ti OR 'less developed population':ab,ti OR 'less developed populations':ab,ti OR 'less developed world':ab,ti OR 'lesser developed country':ab,ti OR 'lesser developed countries':ab,ti OR 'lesser developed nation':ab,ti OR 'lesser developed nations':ab,ti OR 'lesser developed population':ab,ti OR 'lesser developed populations':ab,ti OR 'lesser developed world':ab,ti OR 'under developed country':ab,ti OR 'under developed countries':ab,ti OR 'under developed nation':ab,ti OR 'under developed nations':ab,ti OR 'under developed population':ab,ti OR 'under developed populations':ab,ti OR 'under developed world':ab,ti OR 'underdeveloped country':ab,ti OR 'underdeveloped countries':ab,ti OR 'underdeveloped nation':ab,ti OR 'underdeveloped nations':ab,ti OR 'underdeveloped population':ab,ti OR 'underdeveloped populations':ab,ti OR 'underdeveloped world':ab,ti OR 'middle income country':ab,ti OR 'middle income countries':ab,ti OR 'middle income nation':ab,ti OR 'middle income nations':ab,ti OR 'middle income population':ab,ti OR 'middle income populations':ab,ti OR 'low income country':ab,ti OR 'low income countries':ab,ti OR 'low income nation':ab,ti OR 'low income nations':ab,ti OR 'low income population':ab,ti OR 'low income populations':ab,ti OR 'lower income country':ab,ti OR 'lower income countries':ab,ti OR 'lower income nation':ab,ti OR 'lower income nations':ab,ti OR 'lower income population':ab,ti OR 'lower income populations':ab,ti OR 'underserved country':ab,ti OR 'underserved countries':ab,ti OR 'underserved nation':ab,ti OR 'underserved nations':ab,ti OR 'underserved population':ab,ti OR 'underserved populations':ab,ti OR 'underserved world':ab,ti OR 'under served country':ab,ti OR 'under served countries':ab,ti OR 'under served nation':ab,ti OR 'under served nations':ab,ti OR 'under served population':ab,ti OR 'under served populations':ab,ti OR 'under served world':ab,ti OR 'deprived country':ab,ti OR 'deprived countries':ab,ti OR 'deprived nation':ab,ti OR 'deprived nations':ab,ti OR 'deprived population':ab,ti OR 'deprived populations':ab,ti OR 'deprived world':ab,ti OR 'poor country':ab,ti OR 'poor countries':ab,ti OR 'poor nation':ab,ti OR 'poor nations':ab,ti OR 'poor population':ab,ti OR 'poor populations':ab,ti OR 'poor world':ab,ti OR 'poorer country':ab,ti OR 'poorer countries':ab,ti OR 'poorer nation':ab,ti OR 'poorer nations':ab,ti OR 'poorer population':ab,ti OR 'poorer populations':ab,ti OR 'poorer world':ab,ti OR 'developing economy':ab,ti OR 'developing economies':ab,ti OR 'less developed economy':ab,ti OR 'less developed economies':ab,ti OR 'lesser developed economy':ab,ti OR 'lesser developed economies':ab,ti OR 'under developed economy':ab,ti OR 'under developed economies':ab,ti OR 'underdeveloped economy':ab,ti OR 'underdeveloped economies':ab,ti OR 'middle income economy':ab,ti OR 'middle income economies':ab,ti OR 'low income economy':ab,ti OR 'low income economies':ab,ti OR 'lower income economy':ab,ti OR 'lower income economies':ab,ti OR 'low gdp':ab,ti OR 'low gnp':ab,ti OR 'low gross domestic':ab,ti OR 'low gross national':ab,ti OR 'lower gdp':ab,ti OR 'lower gnp':ab,ti OR 'lower gross domestic':ab,ti OR 'lower gross national':ab,ti OR lmic:ab,ti OR lmics:ab,ti OR 'third world':ab,ti OR 'lami country':ab,ti OR 'lami countries':ab,ti OR 'transitional country':ab,ti OR 'transitional countries':ab,ti

**#2 Search** Africa:ti,ab OR Asia:ti,ab OR Caribbean:ti,ab OR 'West Indies':ti,ab OR 'South America':ti,ab OR 'Latin America':ti,ab OR 'Central America':ti,ab OR 'atlantic islands':ab,ti OR 'commonwealth of independent states':ab,ti OR 'pacific islands':ab,ti OR 'indian ocean islands':ab,ti OR 'eastern europe':ab,ti

**#3 Search** Afghanistan:ti,ab OR Albania:ti,ab OR Algeria:ti,ab OR Angola:ti,ab OR Antigua:ti,ab OR Barbuda:ti,ab OR Argentina:ti,ab OR Armenia:ti,ab OR Armenian:ti,ab OR Aruba:ti,ab OR Azerbaijan:ti,ab OR Bahrain:ti,ab OR Bangladesh:ti,ab OR Barbados:ti,ab OR Benin:ti,ab OR Byelarus:ti,ab OR Byelorussian:ti,ab OR Belarus:ti,ab OR Belorussian:ti,ab OR Belorussia:ti,ab OR Belize:ti,ab OR Bhutan:ti,ab OR Bolivia:ti,ab OR Bosnia:ti,ab OR Herzegovina:ti,ab OR Hercegovina:ti,ab OR Botswana:ti,ab OR Brasil:ti,ab OR Brazil:ti,ab OR Bulgaria:ti,ab OR 'Burkina Faso':ti,ab OR 'Burkina Fasso':ti,ab OR 'Upper Volta':ti,ab OR Burundi:ti,ab OR Urundi:ti,ab OR Cambodia:ti,ab OR 'Khmer Republic':ti,ab OR Kampuchea:ti,ab OR Cameroon:ti,ab OR Cameroons:ti,ab OR Cameron:ti,ab OR Camerons:ti,ab OR 'Cape Verde':ti,ab OR 'Central African Republic':ti,ab OR Chad:ti,ab OR Chile:ti,ab OR China:ti,ab OR Colombia:ti,ab OR Comoros:ti,ab OR 'Comoro Islands':ti,ab OR Comores:ti,ab OR Mayotte:ti,ab OR Congo:ti,ab OR Zaire:ti,ab OR 'Costa Rica':ti,ab OR 'Cote d`Ivoire' OR 'Ivory Coast':ti,ab OR Croatia:ti,ab OR Cuba:ti,ab OR Cyprus:ti,ab OR Czechoslovakia:ti,ab OR 'Czech Republic':ti,ab OR Slovakia:ti,ab OR 'Slovak Republic':ti,ab OR Djibouti:ti,ab OR 'French Somaliland':ti,ab OR Dominica:ti,ab OR 'Dominican Republic':ti,ab OR 'East Timor':ti,ab OR 'East Timur':ti,ab OR 'Timor Leste':ti,ab OR Ecuador:ti,ab OR Egypt:ti,ab OR 'United Arab Republic':ti,ab OR El Salvador:ti,ab OR Eritrea:ti,ab OR Estonia:ti,ab OR Ethiopia:ti,ab OR Fiji:ti,ab OR Gabon:ti,ab OR 'Gabonese Republic':ti,ab OR Gambia:ti,ab OR Gaza:ti,ab OR 'Georgia Republic':ti,ab OR 'Georgian Republic':ti,ab OR Ghana:ti,ab OR 'Gold Coast':ti,ab OR Greece:ti,ab OR Grenada:ti,ab OR Guatemala:ti,ab OR Guinea:ti,ab OR Guam:ti,ab OR Guiana:ti,ab OR Guyana:ti,ab OR Haiti:ti,ab OR Honduras:ti,ab OR Hungary:ti,ab OR India:ti,ab OR Maldives:ti,ab OR Indonesia:ti,ab OR Iran:ti,ab OR Iraq:ti,ab OR 'Isle of Man':ti,ab OR Jamaica:ti,ab OR Jordan:ti,ab OR Kazakhstan:ti,ab OR Kazakh:ti,ab OR Kenya:ti,ab OR Kiribati:ti,ab OR Korea:ti,ab OR Kosovo:ti,ab OR Kyrgyzstan:ti,ab OR Kirghizia:ti,ab OR 'Kyrgyz Republic':ti,ab OR Kirghiz:ti,ab OR Kirgizstan:ti,ab OR 'Lao PDR':ti,ab OR Laos:ti,ab OR Latvia:ti,ab OR Lebanon:ti,ab OR Lesotho:ti,ab OR Basutoland:ti,ab OR Liberia:ti,ab OR Libya:ti,ab OR Lithuania:ti,ab

**#4 Search** Macedonia:ti,ab OR Madagascar:ti,ab OR 'Malagasy Republic':ti,ab OR Malaysia:ti,ab OR Malaya:ti,ab OR Malay:ti,ab OR Sabah:ti,ab OR Sarawak:ti,ab OR Malawi:ti,ab OR Nyasaland:ti,ab OR Mali:ti,ab OR Malta:ti,ab OR 'Marshall Islands':ti,ab OR Mauritania:ti,ab OR Mauritius:ti,ab OR melanesia:ab,ti OR 'Agalega Islands':ti,ab OR Mexico:ti,ab OR Micronesia:ti,ab OR 'Middle East':ti,ab OR Moldova:ti,ab OR Moldovia:ti,ab OR Moldovian:ti,ab OR Mongolia:ti,ab OR Montenegro:ti,ab OR Morocco:ti,ab OR Ifni:ti,ab OR Mozambique:ti,ab OR Myanmar:ti,ab OR Myanma:ti,ab OR Burma:ti,ab OR Namibia:ti,ab OR Nepal:ti,ab OR 'Netherlands Antilles':ti,ab OR 'New Caledonia':ti,ab OR Nicaragua:ti,ab OR Niger:ti,ab OR Nigeria:ti,ab OR 'Northern Mariana Islands':ti,ab OR Oman:ti,ab OR Muscat:ti,ab OR Pakistan:ti,ab OR Palau:ti,ab OR Palestine:ti,ab OR Panama:ti,ab OR Paraguay:ti,ab OR Peru:ti,ab OR Philippines:ti,ab OR Philipines:ti,ab OR Phillipines:ti,ab OR Phillippines:ti,ab OR Poland:ti,ab OR Portugal:ti,ab OR 'Puerto Rico':ti,ab OR Romania:ti,ab OR Rumania:ti,ab OR Roumania:ti,ab OR Russia:ti,ab OR Russian:ti,ab OR Rwanda:ti,ab OR Ruanda:ti,ab OR 'Saint Kitts':ti,ab OR 'St Kitts':ti,ab OR Nevis:ti,ab OR 'Saint Lucia':ti,ab OR 'St Lucia':ti,ab OR 'Saint Vincent':ti,ab OR 'St Vincent':ti,ab OR Grenadines:ti,ab OR Samoa:ti,ab OR 'Samoan Islands':ti,ab OR 'Navigator Island':ti,ab OR 'Navigator Islands':ti,ab OR 'Sao Tome':ti,ab OR 'Saudi Arabia':ti,ab OR Senegal:ti,ab OR Serbia:ti,ab OR Montenegro:ti,ab OR Seychelles:ti,ab OR 'Sierra Leone':ti,ab OR Slovenia:ti,ab OR 'Sri Lanka':ti,ab OR Ceylon:ti,ab OR 'Solomon Islands':ti,ab OR Somalia:ti,ab OR Sudan:ti,ab OR Suriname:ti,ab OR Surinam:ti,ab OR Swaziland:ti,ab OR Syria:ti,ab OR Syrian:ti,ab OR Tajikistan:ti,ab OR Tadzhikistan:ti,ab OR Tadjikistan:ti,ab OR Tadzhik:ti,ab OR Tanzania:ti,ab OR Thailand:ti,ab OR Togo:ti,ab OR 'Togolese Republic':ti,ab OR Tonga:ti,ab OR Trinidad:ti,ab OR Tobago:ti,ab OR Tunisia:ti,ab OR Turkey:ti,ab OR Turkmenistan:ti,ab OR Turkmen:ti,ab OR Tuvalu:ti,ab OR Uganda:ti,ab OR Ukraine:ti,ab OR Uruguay:ti,ab OR USSR:ti,ab OR 'Soviet Union':ti,ab OR 'Union of Soviet Socialist Republics':ti,ab OR Uzbekistan:ti,ab OR Uzbek OR Vanuatu:ti,ab OR 'New Hebrides':ti,ab OR Venezuela:ti,ab OR Vietnam:ti,ab OR 'Viet Nam':ti,ab OR 'West Bank':ti,ab OR Yemen:ti,ab OR Yugoslavia:ti,ab OR Zambia:ti,ab OR Zimbabwe:ti,ab OR Rhodesia:ti,ab

**#5 Search:** 'developing country'/exp OR 'Africa'/de OR 'Africa south of the Sahara'/de OR 'North Africa'/de OR 'Central Africa'/de OR 'Asia'/de OR 'South Asia'/de OR 'Southeast Asia'/de OR 'South America'/de OR 'Central America'/de OR 'South and Central America'/de OR 'Atlantic islands'/de OR 'Caribbean Islands'/de OR 'Pacific islands'/de OR 'Indian Ocean'/de OR 'Eastern Europe'/de

**#6 Search** Afghanistan/exp OR Albania/exp OR Algeria/exp OR 'American Samoa'/exp OR Angola/exp OR 'Antigua and Barbuda'/exp OR Argentina/exp OR Armenia/exp OR Azerbaijan/exp OR Bahrain/exp OR Bangladesh/exp OR Barbados/exp OR Benin/exp OR 'Belarus'/exp OR 'Baltic States'/exp OR Belize/exp OR Bhutan/exp OR Bolivia/exp OR 'Bosnia and Herzegovina'/exp OR Botswana/exp OR Brazil/exp OR Bulgaria/exp OR 'Burkina Faso'/exp OR Burundi/exp OR Cambodia/exp OR Cameroon/exp OR 'Cape Verde'/exp OR 'Central African Republic'/exp OR Chad/exp OR Chile/exp OR China/exp OR Colombia/exp OR Comoros/exp OR Congo/exp OR 'Costa Rica'/exp OR 'Cote d`Ivoire'/exp OR Croatia/exp OR Cuba/exp OR Cyprus/exp OR Czechoslovakia/exp OR 'Czech Republic'/exp OR Slovakia/exp OR Djibouti/exp OR 'Democratic Republic Congo'/exp OR Dominica/exp OR 'Dominican Republic'/exp OR 'Timor-Leste'/exp OR Ecuador/exp OR Egypt/exp OR 'El Salvador'/exp OR Eritrea/exp OR Estonia/exp OR Ethiopia/exp OR 'French Guiana'/exp OR Fiji/exp OR Gabon/exp OR Gambia/exp OR 'Georgia (Republic) '/exp OR Ghana/exp OR Greece/exp OR Grenada/exp OR Guatemala/exp OR Guinea/exp OR Guinea-Bissau/exp OR Guam/exp OR Guyana/exp OR Haiti/exp OR Honduras/exp OR Hungary/exp OR India/exp OR Indonesia/exp OR Iran/exp OR Iraq/exp OR Jamaica/exp OR Jordan/exp OR Kazakhstan/exp OR Kenya/exp OR Korea/exp OR Kyrgyzstan/exp OR Laos/exp OR Latvia/exp OR Lebanon/exp OR Lesotho/exp OR Liberia/exp OR 'Libyan Arab Jamahiriya'/exp OR Lithuania/exp OR 'Macedonia (republic)'/exp OR Madagascar/exp OR Malaysia/exp OR Malawi/exp OR Mali/exp OR Malta/exp OR Mauritania/exp OR Mauritius/exp OR "Melanesia"/exp

**#7 Search** Mexico/exp OR 'Federated States of Micronesia'/exp OR 'Middle East'/de OR Moldova/exp OR Mongolia/exp OR Montenegro/exp OR Morocco/exp OR Mozambique/exp OR Myanmar/exp OR Namibia/exp OR Nepal/exp OR 'Netherlands Antilles'/exp OR 'New Caledonia'/exp OR Nicaragua/exp OR Niger/exp OR Nigeria/exp OR 'North Korea'/exp OR Oman/exp OR Pakistan/exp OR Palau/exp OR Panama/exp OR 'Papua New Guinea'/exp OR Paraguay/exp OR Peru/exp OR Philippines/exp OR Poland/exp OR Portugal/exp OR 'Puerto Rico'/exp OR Romania/exp OR 'Russian Federation'/exp OR Rwanda/exp OR 'Saint Kitts and Nevis'/exp OR 'Saint Lucia'/exp OR 'Saint Vincent and the Grenadines'/exp OR 'Samoan Islands'/exp OR Samoa/exp OR 'Saudi Arabia'/exp OR Senegal/exp OR Serbia/exp OR 'Montenegro (republic)'/exp OR Seychelles/exp OR 'Sierra Leone'/exp OR Slovenia/exp OR 'Sri Lanka'/exp OR Somalia/exp OR 'South Korea'/exp OR 'South Africa'/exp OR Sudan/exp OR Suriname/exp OR Swaziland/exp OR 'Syrian Arab Republic'/exp OR Tajikistan/exp OR Tanzania/exp OR Thailand/exp OR Togo/exp OR Tonga/exp OR 'Trinidad and Tobago'/exp OR Tunisia/exp OR 'Turkey (republic)'/exp OR Turkmenistan/exp OR Uganda/exp OR Ukraine/exp OR Uruguay/exp OR USSR/exp OR Uzbekistan/exp OR Vanuatu/exp OR Venezuela/exp OR 'Viet Nam'/exp OR Yemen/exp OR Yugoslavia/exp OR 'Yugoslavia (pre-1992)'/exp OR Zambia/exp OR Zimbabwe/exp

Scopus search strategy:

TITLE-ABS-KEY({Emergency medicine} OR {Emergency services} OR {Emergency department} OR {Emergency service} OR {Emergency departments} OR {ER} OR {ED} OR {Emergency room} OR {Emergency rooms} OR {Emergency ward} OR {Emergency Unit} OR {Trauma Center} OR {Trauma Centers} OR {emergency health service} OR {emergency health services} OR {emergency medical services} OR {emergency medical service} OR {accident and emergency} OR {accident & emergency} OR {a&e} OR {A & E})

AND

TITLE-ABS-KEY({HIV} OR {HIV-1} OR {HIV-2} OR {human immunodeficiency virus} OR {human immunodeficiency viruses} OR {HTLV-III} OR {human T cell lymphotrophic virus type III} OR {human T lymphotropic Virus type III} OR {human t-cell leukemia virus type III} OR {human t cell leukemia virus type III} OR {human t-cell lymphotropic virus type III} OR {human t-lymphotropic virus type III} OR {LAV-HTLV-III} OR {lymphadenopathy-associated virus} OR {lymphadenopathy associated virus} OR {lymphadenopathy-associated viruses} OR {lymphadenopathy associated virus} OR {Acquired Immunodeficiency Syndrome} OR {AIDS} OR {acquired immunologic deficiency syndrome} OR {acquired immune deficiency syndrome} OR {acquired immuno-deficiency syndrome} OR {acquired immuno deficiency syndrome} OR {acquired immuno-deficiency syndromes} OR {acquired immuno deficiency syndromes} OR {acquired immunodeficiency syndromes})

AND

**Combine #1 OR #2 OR #3 OR #4**

**#1 Search** TITLE-ABS-KEY({developing country} OR {developing countries} OR {developing nation} OR {developing nations} OR {developing population} OR {developing populations} OR {developing world} OR {less developed country} OR {less developed countries} OR {less developed nation} OR {less developed nations} OR {less developed population} OR {less developed populations} OR {less developed world} OR {lesser developed country} OR {lesser developed countries} OR {lesser developed nation} OR {lesser developed nations} OR {lesser developed population} OR {lesser developed populations} OR {lesser developed world} OR {under developed country} OR {under developed countries} OR {under developed nation} OR {under developed nations} OR {under developed population} OR {under developed populations} OR {under developed world} OR {underdeveloped country} OR {underdeveloped countries} OR {underdeveloped nation} OR {underdeveloped nations} OR {underdeveloped population} OR {underdeveloped populations} OR {underdeveloped world} OR {middle income country} OR {middle income countries} OR {middle income nation} OR {middle income nations} OR {middle income population} OR {middle income populations} OR {low income country} OR {low income countries} OR {low income nation} OR {low income nations} OR {low income population} OR {low income populations} OR {lower income country} OR {lower income countries} OR {lower income nation} OR {lower income nations} OR {lower income population} OR {lower income populations} OR {underserved country} OR {underserved countries} OR {underserved nation} OR {underserved nations} OR {underserved population} OR {underserved populations} OR {underserved world} OR {under served country} OR {under served countries} OR {under served nation} OR {under served nations} OR {under served population} OR {under served populations} OR {under served world} OR {deprived country} OR {deprived countries} OR {deprived nation} OR {deprived nations} OR {deprived population} OR {deprived populations} OR {deprived world})

**#2 Search** TITLE-ABS-KEY({poor country} OR {poor countries} OR {poor nation} OR {poor nations} OR {poor population} OR {poor populations} OR {poor world} OR {poorer country} OR {poorer countries} OR {poorer nation} OR {poorer nations} OR {poorer population} OR {poorer populations} OR {poorer world} OR {developing economy} OR {developing economies} OR {less developed economy} OR {less developed economies} OR {lesser developed economy} OR {lesser developed economies} OR {under developed economy} OR {under developed economies} OR {underdeveloped economy} OR {underdeveloped economies} OR {middle income economy} OR {middle income economies} OR {low income economy} OR {low income economies} OR {lower income economy} OR {lower income economies} OR {low gdp} OR {low gnp} OR {low gross domestic} OR {low gross national} OR {lower gdp} OR {lower gnp} OR {lower gross domestic} OR {lower gross national} OR lmic OR lmics OR {third world} OR {lami country} OR {lami countries} OR {transitional country} OR {transitional countries})

**#3 Search** TITLE-ABS-KEY(Africa OR Asia OR Caribbean OR {West Indies} OR {South America} OR {Latin America} OR {Central America} OR {Atlantic Islands} OR {Commonwealth of Independent States} OR {Pacific Islands} OR {Indian Ocean Islands} OR {Eastern Europe} OR Afghanistan OR Albania OR Algeria OR Angola OR Antigua OR Barbuda OR Argentina OR Armenia OR Armenian OR Aruba OR Azerbaijan OR Bahrain OR Bangladesh OR Barbados OR Benin OR Byelarus OR Byelorussian OR Belarus OR Belorussian OR Belorussia OR Belize OR Bhutan OR Bolivia OR Bosnia OR Herzegovina OR Hercegovina OR Botswana OR Brasil OR Brazil OR Bulgaria OR {Burkina Faso} OR {Burkina Fasso} OR {Upper Volta} OR Burundi OR Urundi OR Cambodia OR {Khmer Republic} OR Kampuchea OR Cameroon OR Cameroons OR Cameron OR Camerons OR {Cape Verde} OR {Central African Republic} OR Chad OR Chile OR China OR Colombia OR Comoros OR {Comoro Islands} OR Comores OR Mayotte OR Congo OR Zaire OR {Costa Rica} OR {Cote d'Ivoire} OR {Ivory Coast} OR Croatia OR Cuba OR Cyprus OR Czechoslovakia OR {Czech Republic} OR Slovakia OR {Slovak Republic} OR Djibouti OR {French Somaliland} OR Dominica OR {Dominican Republic} OR {East Timor} OR {East Timur} OR {Timor Leste} OR Ecuador OR Egypt OR {United Arab Republic} OR {El Salvador} OR Eritrea OR Estonia OR Ethiopia OR Fiji OR Gabon OR {Gabonese Republic} OR Gambia OR Gaza OR {Georgia Republic} OR {Georgian Republic} OR Ghana OR {Gold Coast} OR Greece OR Grenada OR Guatemala OR Guinea OR Guam OR Guiana OR Guyana OR Haiti OR Honduras OR Hungary OR India OR Maldives OR Indonesia OR Iran OR Iraq OR {Isle of Man} OR Jamaica OR Jordan OR Kazakhstan OR Kazakh OR Kenya OR Kiribati OR Korea OR Kosovo OR Kyrgyzstan OR Kirghizia OR {Kyrgyz Republic} OR Kirghiz OR Kirgizstan OR {Lao PDR} OR Laos OR Latvia OR Lebanon OR Lesotho OR Basutoland OR Liberia OR Libya OR Lithuania)

**#4 Search** TITLE-ABS-KEY(Macedonia OR Madagascar OR {Malagasy Republic} OR Malaysia OR Malaya OR Malay OR Sabah OR Sarawak OR Malawi OR Nyasaland OR Mali OR Malta OR {Marshall Islands} OR Mauritania OR Mauritius OR {Agalega Islands} OR {Melanesia} OR Mexico OR Micronesia OR {Middle East} OR Moldova OR Moldovia OR Moldovian OR Mongolia OR Montenegro OR Morocco OR Ifni OR Mozambique OR Myanmar OR Myanma OR Burma OR Namibia OR Nepal OR {Netherlands Antilles} OR {New Caledonia} OR Nicaragua OR Niger OR Nigeria OR {Northern Mariana Islands} OR Oman OR Muscat OR Pakistan OR Palau OR Palestine OR Panama OR Paraguay OR Peru OR Philippines OR Philipines OR Phillipines OR Phillippines OR Poland OR Portugal OR {Puerto Rico} OR Romania OR Rumania OR Roumania OR Russia OR Russian OR Rwanda OR Ruanda OR {Saint Kitts} OR {St Kitts} OR Nevis OR {Saint Lucia} OR {St Lucia} OR {Saint Vincent} OR {St Vincent} OR Grenadines OR Samoa OR {Samoan Islands} OR {Navigator Island} OR {Navigator Islands} OR {Sao Tome} OR {Saudi Arabia} OR Senegal OR Serbia OR Montenegro OR Seychelles OR {Sierra Leone} OR Slovenia OR {Sri Lanka} OR Ceylon OR {Solomon Islands} OR Somalia OR Sudan OR Suriname OR Surinam OR Swaziland OR Syria OR Syrian OR Tajikistan OR Tadzhikistan OR Tadjikistan OR Tadzhik OR Tanzania OR Thailand OR Togo OR {Togolese Republic} OR Tonga OR Trinidad OR Tobago OR Tunisia OR Turkey OR Turkmenistan OR Turkmen OR Tuvalu OR Uganda OR Ukraine OR Uruguay OR USSR OR {Soviet Union} OR {Union of Soviet Socialist Republics} OR Uzbekistan OR Uzbek OR Vanuatu OR {New Hebrides} OR Venezuela OR Vietnam OR {Viet Nam} OR {West Bank} OR Yemen OR Yugoslavia OR Zambia OR Zimbabwe OR Rhodesia)

Web of Science search strategy:

TS=("Emergency medicine" OR "Emergency services" OR "Emergency department" OR "Emergency service" OR "Emergency departments" OR "ER" OR "ED" OR "Emergency room" OR "Emergency rooms" OR "Emergency ward" OR "Emergency Unit" OR "Trauma Center" OR "Trauma Centers" OR “emergency health service” OR “emergency health services” OR “emergency medical services” OR “emergency medical service” OR "accident and emergency" OR "accident & emergency" OR "a&e" OR "A & E")

AND

TS=("HIV" OR "HIV-1" OR "HIV-2" OR "human immunodeficiency virus" OR "human immunodeficiency viruses" OR "HTLV-III" OR "human T cell lymphotrophic virus type III" OR "human T lymphotropic Virus type III" OR "human t-cell leukemia virus type III" OR "human t cell leukemia virus type III" OR "human t-cell lymphotropic virus type III" OR "human t-lymphotropic virus type III" OR "LAV-HTLV-III" OR "lymphadenopathy-associated virus" OR "lymphadenopathy associated virus" OR "lymphadenopathy-associated viruses" OR "lymphadenopathy associated virus" OR "Acquired Immunodeficiency Syndrome" OR "AIDS" OR "acquired immunologic deficiency syndrome" OR "acquired immune deficiency syndrome" OR "acquired immuno-deficiency syndrome" OR "acquired immuno deficiency syndrome" OR "acquired immuno-deficiency syndromes" OR "acquired immuno deficiency syndromes" OR "acquired immunodeficiency syndromes")

AND

**Combine: (#1 OR #2 OR #3 OR #4)**

**#1 Search** TS=("developing country" OR "developing countries" OR "developing nation" OR "developing nations" OR "developing population" OR "developing populations" OR "developing world" OR "less developed country" OR "less developed countries" OR "less developed nation" OR "less developed nations" OR "less developed population" OR "less developed populations" OR "less developed world" OR "lesser developed country" OR "lesser developed countries" OR "lesser developed nation" OR "lesser developed nations" OR "lesser developed population" OR "lesser developed populations" OR "lesser developed world" OR "under developed country" OR "under developed countries" OR "under developed nation" OR "under developed nations" OR "under developed population" OR "under developed populations" OR "under developed world" OR "underdeveloped country" OR "underdeveloped countries" OR "underdeveloped nation" OR "underdeveloped nations" OR "underdeveloped population" OR "underdeveloped populations" OR "underdeveloped world" OR "middle income country" OR "middle income countries" OR "middle income nation" OR "middle income nations" OR "middle income population" OR "middle income populations" OR "low income country" OR "low income countries" OR "low income nation" OR "low income nations" OR "low income population" OR "low income populations" OR "lower income country" OR "lower income countries" OR "lower income nation" OR "lower income nations" OR "lower income population" OR "lower income populations" OR "underserved country" OR "underserved countries" OR "underserved nation" OR "underserved nations" OR "underserved population" OR "underserved populations" OR "underserved world" OR "under served country" OR "under served countries" OR "under served nation" OR "under served nations" OR "under served population" OR "under served populations" OR "under served world" OR "deprived country" OR "deprived countries" OR "deprived nation" OR "deprived nations" OR "deprived population" OR "deprived populations" OR "deprived world")

**#2 Search** TS=("poor country" OR "poor countries" OR "poor nation" OR "poor nations" OR "poor population" OR "poor populations" OR "poor world" OR "poorer country" OR "poorer countries" OR "poorer nation" OR "poorer nations" OR "poorer population" OR "poorer populations" OR "poorer world" OR "developing economy" OR "developing economies" OR "less developed economy" OR "less developed economies" OR "lesser developed economy" OR "lesser developed economies" OR "under developed economy" OR "under developed economies" OR "underdeveloped economy" OR "underdeveloped economies" OR "middle income economy" OR "middle income economies" OR "low income economy" OR "low income economies" OR "lower income economy" OR "lower income economies" OR "low gdp" OR "low gnp" OR "low gross domestic" OR "low gross national" OR "lower gdp" OR "lower gnp" OR "lower gross domestic" OR "lower gross national" OR lmic OR lmics OR "third world" OR "lami country" OR "lami countries" OR "transitional country" OR "transitional countries")

**#3 Search** TS=(Africa OR Asia OR Caribbean OR "West Indies" OR "South America" OR "Latin America" OR "Central America" OR "Atlantic Islands" OR "Commonwealth of Independent States" OR "Pacific Islands" OR "Indian Ocean Islands" OR "Eastern Europe" OR Afghanistan OR Albania OR Algeria OR Angola OR Antigua OR Barbuda OR Argentina OR Armenia OR Armenian OR Aruba OR Azerbaijan OR Bahrain OR Bangladesh OR Barbados OR Benin OR Byelarus OR Byelorussian OR Belarus OR Belorussian OR Belorussia OR Belize OR Bhutan OR Bolivia OR Bosnia OR Herzegovina OR Hercegovina OR Botswana OR Brasil OR Brazil OR Bulgaria OR "Burkina Faso" OR "Burkina Fasso" OR "Upper Volta" OR Burundi OR Urundi OR Cambodia OR "Khmer Republic" OR Kampuchea OR Cameroon OR Cameroons OR Cameron OR Camerons OR "Cape Verde" OR "Central African Republic" OR Chad OR Chile OR China OR Colombia OR Comoros OR "Comoro Islands" OR Comores OR Mayotte OR Congo OR Zaire OR "Costa Rica" OR "Cote d'Ivoire" OR "Ivory Coast" OR Croatia OR Cuba OR Cyprus OR Czechoslovakia OR "Czech Republic" OR Slovakia OR "Slovak Republic" OR Djibouti OR "French Somaliland" OR Dominica OR "Dominican Republic" OR "East Timor" OR "East Timur" OR "Timor Leste" OR Ecuador OR Egypt OR "United Arab Republic" OR "El Salvador" OR Eritrea OR Estonia OR Ethiopia OR Fiji OR Gabon OR "Gabonese Republic" OR Gambia OR Gaza OR "Georgia Republic" OR "Georgian Republic" OR Ghana OR "Gold Coast" OR Greece OR Grenada OR Guatemala OR Guinea OR Guam OR Guiana OR Guyana OR Haiti OR Honduras OR Hungary OR India OR Maldives OR Indonesia OR Iran OR Iraq OR "Isle of Man" OR Jamaica OR Jordan OR Kazakhstan OR Kazakh OR Kenya OR Kiribati OR Korea OR Kosovo OR Kyrgyzstan OR Kirghizia OR "Kyrgyz Republic" OR Kirghiz OR Kirgizstan OR "Lao PDR" OR Laos OR Latvia OR Lebanon OR Lesotho OR Basutoland OR Liberia OR Libya OR Lithuania)

**#4 Search** TS=(Macedonia OR Madagascar OR "Malagasy Republic" OR Malaysia OR Malaya OR Malay OR Sabah OR Sarawak OR Malawi OR Nyasaland OR Mali OR Malta OR "Marshall Islands" OR Mauritania OR Mauritius OR "Agalega Islands" OR "Melanesia" OR Mexico OR Micronesia OR "Middle East" OR Moldova OR Moldovia OR Moldovian OR Mongolia OR Montenegro OR Morocco OR Ifni OR Mozambique OR Myanmar OR Myanma OR Burma OR Namibia OR Nepal OR "Netherlands Antilles" OR "New Caledonia" OR Nicaragua OR Niger OR Nigeria OR "Northern Mariana Islands" OR Oman OR Muscat OR Pakistan OR Palau OR Palestine OR Panama OR Paraguay OR Peru OR Philippines OR Philipines OR Phillipines OR Phillippines OR Poland OR Portugal OR "Puerto Rico" OR Romania OR Rumania OR Roumania OR Russia OR Russian OR Rwanda OR Ruanda OR "Saint Kitts" OR "St Kitts" OR Nevis OR "Saint Lucia" OR "St Lucia" OR "Saint Vincent" OR "St Vincent" OR Grenadines OR Samoa OR "Samoan Islands" OR "Navigator Island" OR "Navigator Islands" OR "Sao Tome" OR "Saudi Arabia" OR Senegal OR Serbia OR Montenegro OR Seychelles OR" Sierra Leone" OR Slovenia OR "Sri Lanka" OR Ceylon OR "Solomon Islands" OR Somalia OR Sudan OR Suriname OR Surinam OR Swaziland OR Syria OR Syrian OR Tajikistan OR Tadzhikistan OR Tadjikistan OR Tadzhik OR Tanzania OR Thailand OR Togo OR "Togolese Republic" OR Tonga OR Trinidad OR Tobago OR Tunisia OR Turkey OR Turkmenistan OR Turkmen OR Tuvalu OR Uganda OR Ukraine OR Uruguay OR USSR OR "Soviet Union" OR "Union of Soviet Socialist Republics" OR Uzbekistan OR Uzbek OR Vanuatu OR "New Hebrides" OR Venezuela OR Vietnam OR "Viet Nam" OR "West Bank" OR Yemen OR Yugoslavia OR Zambia OR Zimbabwe OR Rhodesia)

Cochrane search strategy:

HIV/AIDS MeSH:

HIV

HIV-1

HIV-2

Acquired Immunodeficiency Syndrome

HIV/AIDS keywords:

"HIV":ti,ab,kw OR "HIV-1":ti,ab,kw OR "HIV-2":ti,ab,kw OR "human immunodeficiency virus":ti,ab,kw OR "human immunodeficiency virus 1":ti,ab,kw OR "human immunodeficiency virus 2":ti,ab,kw OR "human immunodeficiency viruses":ti,ab,kw OR "HTLV-III":ti,ab,kw OR "human T cell lymphotrophic virus type III":ti,ab,kw OR "human T lymphotropic Virus type III":ti,ab,kw OR "human t-cell leukemia virus type III":ti,ab,kw OR "human t cell leukemia virus type III":ti,ab,kw OR "human t-cell lymphotropic virus type III":ti,ab,kw OR "human t-lymphotropic virus type III":ti,ab,kw OR "LAV-HTLV-III":ti,ab,kw OR "lymphadenopathy-associated virus":ti,ab,kw OR "lymphadenopathy associated virus":ti,ab,kw OR "lymphadenopathy-associated viruses":ti,ab,kw OR "lymphadenopathy associated virus":ti,ab,kw OR "acquired immunodeficiency syndrome":ti,ab,kw OR "AIDS":ti,ab,kw OR "acquired immunologic deficiency syndrome":ti,ab,kw OR "acquired immune deficiency syndrome":ti,ab,kw OR "acquired immuno-deficiency syndrome":ti,ab,kw OR "acquired immuno deficiency syndrome":ti,ab,kw OR "acquired immuno-deficiency syndromes":ti,ab,kw OR "acquired immuno deficiency syndromes":ti,ab,kw OR "acquired immunodeficiency syndromes":ti,ab,kw

AND

Emergency medicine MeSH:

Emergency Service, Hospital

Emergency Medicine

Trauma Centers

Emergency Medicine Keywords:

“Emergency medicine”:ti,ab,kw OR “Emergency services”:ti,ab,kw OR “Emergency department”:ti,ab,kw OR “Emergency service”:ti,ab,kw OR “Emergency departments”:ti,ab,kw OR “ER”:ti,ab,kw OR "ED":ti,ab,kw OR “Emergency room”:ti,ab,kw OR “Emergency rooms”:ti,ab,kw OR “Emergency ward”:ti,ab,kw OR “Emergency Unit”:ti,ab,kw OR “Trauma Center”:ti,ab,kw OR “Trauma Centers”:ti,ab,kw OR “emergency health service”:ti,ab,kw OR “emergency health services”:ti,ab,kw OR “emergency medical services”:ti,ab,kw OR “emergency medical service”:ti,ab,kw OR "accident & emergency":ti,ab,kw OR "accident and emergency":ti,ab,kw OR "A&E":ti,ab,kw OR "A & E":ti,ab,kw

AND

**#1** (Africa or Asia or Caribbean or "West Indies" or "South America" or "Latin America" or "Central America")**:ti,ab,kw**

**#2** (Afghanistan or Albania or Algeria or Angola or Antigua or Barbuda or Argentina or Armenia or Armenian or Aruba or Azerbaijan or Bahrain or Bangladesh or Barbados or Benin or Byelarus or Byelorussian or Belarus or Belorussian or Belorussia or Belize or Bhutan or Bolivia or Bosnia or Herzegovina or Hercegovina or Botswana or Brasil or Brazil or Bulgaria or "Burkina Faso" or "Burkina Fasso" or "Upper Volta" or Burundi or Urundi or Cambodia or "Khmer Republic" or Kampuchea or Cameroon or Cameroons or Cameron or Camerons or "Cape Verde" or "Central African Republic" or Chad or Chile or China or Colombia or Comoros or "Comoro Islands" or Comores or Mayotte or Congo or Zaire or "Costa Rica" or "Cote d'Ivoire" or "Ivory Coast" or Croatia or Cuba or Cyprus or Czechoslovakia or "Czech Republic" or Slovakia or "Slovak Republic")**:ti,ab,kw**

**#3** (Djibouti or "French Somaliland" or Dominica or "Dominican Republic" or "East Timor" or "East Timur" or "Timor Leste" or Ecuador or Egypt or "United Arab Republic" or "El Salvador" or Eritrea or Estonia or Ethiopia or Fiji or Gabon or "Gabonese Republic" or Gambia or Gaza or Georgia or Georgian or Ghana or "Gold Coast" or Greece or Grenada or Guatemala or Guinea or Guam or Guiana or Guyana or Haiti or Honduras or Hungary or India or Maldives or Indonesia or Iran or Iraq or "Isle of Man" or Jamaica or Jordan or Kazakhstan or Kazakh or Kenya or Kiribati or Korea or Kosovo or Kyrgyzstan or Kirghizia or "Kyrgyz Republic" or Kirghiz or Kirgizstan or "Lao PDR" or Laos or Latvia or Lebanon or Lesotho or Basutoland or Liberia or Libya or Lithuania)**:ti,ab,kw**

**#4** (Macedonia or Madagascar or "Malagasy Republic" or Malaysia or Malaya or Malay or Sabah or Sarawak or Malawi or Nyasaland or Mali or Malta or "Marshall Islands" or Mauritania or Mauritius or "Agalega Islands" or Mexico or Micronesia or "Middle East" or Moldova or Moldovia or Moldovian or Mongolia or Montenegro or Morocco or Ifni or Mozambique or Myanmar or Myanma or Burma or Namibia or Nepal or "Netherlands Antilles" or "New Caledonia" or Nicaragua or Niger or Nigeria or "Northern Mariana Islands" or Oman or Muscat or Pakistan or Palau or Palestine or Panama or Paraguay or Peru or Philippines or Philipines or Phillipines or Phillippines or Poland or Portugal or "Puerto Rico")**:ti,ab,kw**

**#5** (Romania or Rumania or Roumania or Russia or Russian or Rwanda or Ruanda or "Saint Kitts" or "St Kitts" or Nevis or "Saint Lucia" or "St Lucia" or "Saint Vincent" or "St Vincent" or Grenadines or Samoa or "Samoan Islands" or "Navigator Island" or "Navigator Islands" or "Sao Tome" or "Saudi Arabia" or Senegal or Serbia or Montenegro or Seychelles or "Sierra Leone" or Slovenia or "Sri Lanka" or Ceylon or "Solomon Islands" or Somalia or Sudan or Suriname or Surinam or Swaziland or Syria or Tajikistan or Tadzhikistan or Tadjikistan or Tadzhik or Tanzania or Thailand or Togo or "Togolese Republic" or Tonga or Trinidad or Tobago or Tunisia or Turkey or Turkmenistan or Turkmen or Uganda or Ukraine or Uruguay or USSR or "Soviet Union" or "Union of Soviet Socialist Republics" or Uzbekistan or Uzbek or Vanuatu or "New Hebrides" or Venezuela or Vietnam or "Viet Nam" or "West Bank" or Yemen or Yugoslavia or Zambia or Zimbabwe or Rhodesia)**:ti,ab,kw**

**#6** (developing or less* NEXT developed or "under developed" or underdeveloped or "middle income" or low* NEXT income or underserved or "under served" or deprived or poor*) NEXT (countr* or nation* or population* or world)**:ti,ab,kw**

**#7** (developing or less* NEXT developed or "under developed" or underdeveloped or "middle income" or low* NEXT income) NEXT (economy or economies)**:ti,ab,kw**

**#8** low* NEXT (gdp or gnp or "gross domestic" or "gross national")**:ti,ab,kw**

**#9** (low NEAR/3 middle NEAR/3 countr*)**:ti,ab,kw**

**#10** (lmic or lmics or "third world" or "lami country" or "lami countries")**:ti,ab,kw**

**#11** ("transitional country" or "transitional countries")**:ti,ab,kw**

**#12** (#1 OR #2 OR #3 OR #4 OR #5 OR #6 OR #7 OR #8 OR #9 OR #10 OR #11)
